# Raman spectroscopy and bioinformatics-based identification of key genes and pathways capable of distinguishing between diffuse large B cell lymphoma and chronic lymphocytic leukemia

**DOI:** 10.3389/fimmu.2025.1516946

**Published:** 2025-02-25

**Authors:** Haoyue Liang, Zhijie Cao, Yansong Ren, Yihan Li, Haoyu Wang, Fanfan Sun, Mei Xue, Guoqing Zhu, Yuan Zhou

**Affiliations:** ^1^ State Key Laboratory of Experimental Hematology, National Clinical Research Center for Blood Diseases, Haihe Laboratory of Cell Ecosystem, Institute of Hematology & Blood Diseases Hospital, Chinese Academy of Medical Sciences & Peking Union Medical College, Tianjin, China; ^2^ Tianjin Institutes of Health Science, Tianjin, China; ^3^ School of Medical Technology, Tianjin University of Traditional Chinese Medicine, Tianjin, China

**Keywords:** DLBCL, CLL, bioinformatics analysis, Raman spectroscopy, protein-protein interaction networks

## Abstract

Diffuse large B-cell lymphoma (DLBCL) and chronic lymphocytic leukemia (CLL) are subtypes of non-Hogkin lymphoma (NHL) that are generally distinct form one cases, but the transformation of one of these diseases into the other is possible. Some patients with CLL, for instance, have the potential to develop Richter transformation such that they are diagnosed with a rare, invasive DLBCL subtype. In this study, bioinformatics analyses of these two NHL subtypes were conducted, identifying key patterns of gene expression and then experimentally validating the results. Disease-related gene expression datasets from the GEO database were used to identify differentially expressed genes (DEGs) and DEG functions were examined using GO analysis and protein-protein interaction network construction. This strategy revealed many up- and down-regulated DEGs, with functional enrichment analyses identifying these genes as being closely associated with inflammatory and immune response activity. PPI network analyses and the evaluation of clustered network modules indicated the top 10 up- and down-regulated genes involved in disease onset and development. Serological analyses revealed significantly higher ALB, TT, and WBC levels in CLL patients relative to DLBCL patients, whereas the opposite was true with respect to TG, HDL, GGT, ALP, ALT, and NEUT% levels. In comparison to the CLL and DLBCL groups, the healthy control samples demonstrated higher signals of protein peak positions (621, 643, 848, 853, 869, 935, 1003, 1031, 1221, 1230, 1260, 1344, 1443, 1446, 1548, 1579, 1603, 1647 cm^-1^), nucleic acid peak positions (726, 781, 786, 1078, 1190, 1415, 1573, 1579 cm^-1^), beta carotene peak positions (957, 1155, 1162 cm^-1^), carbohydrate peak positions (842 cm^-1^), collagen peak positions (1345 cm^-1^), and lipid peak positions (957, 1078, 1119, 1285, 1299, 1437, 1443, 1446 cm^-1^) compared to the CLL and DLBCL groups. Verification of these key genes in patient samples yielded results consistent with findings derived from bioinformatics analyses, highlighting their relevance to diagnosing and treating these forms of NHL. Together, these analyses identified genes and pathways involved in both DLBCL and CLL. The set of molecular markers established herein can aid in patient diagnosis and prognostic evaluation, providing a valuable foundation for their therapeutic application.

## Introduction

Diffuse large B-cell lymphoma (DLBCL) and chronic lymphocytic leukemia (CLL) are both subtypes of non-Hodgkin lymphoma (NHL). DLBCL, the most common form of NHL, is characterized by lymph node enlargement and potential organ invasion. DLBCL cases originate from mature B cells of germinal center or non-germinal center origin (1–3). The classification of DLBCL cases is based on the biological features of these cells of origin and the overall clinical disease course. ABC-DLBCL, for instance, has a poorer prognosis than GCB-DLBCL. In ABC-DLBCL tumor cells, prolonged B cell receptor (BCR) signaling leads to high levels of NF-κB pathway activity, whereas in GCB-DLBCL tumor cells, BCR signaling contributes to high levels of PI3K signaling activity (4, 5). The characteristics of DLBCL tumor cells influence both tumor progression and overall host immune function. Certain proteins expressed specifically within the central nervous system (CNS) can reportedly bind to the BCRs of DLBCL tumor cells, contributing to oncogenic progression (6). In one study, a bias toward IGV rearrangement was noted in PCNSL tumor cells, with the particular prioritization of IGHV4-34, enabling the recognition of galectin-3 expression on various CNS cells (7). Kawano et al. (8) described a series of primary adrenal DLBCL cases, among which 9 were positive for EBV and 3 had a history of tumors. This provides further support for potential excessive B cell infiltration in particular immunological niches in the context of chemotherapy-induced cancer-related immune dysfunction, as has been reported in EBVMCU cases (9). In their analysis of 62 primary intestinal DLBCL cases, Ishikawa et al. identified 10 cases that were EBV-positive, almost all of which presented with features of immunosuppression and clinical and pathological features overlapping with those of immunodeficient PCNSL (10). In stark contrast, cases of primary gastric EBV-positive DLBCL often present even without any apparent evidence of immunosuppression (11, 12). While the pathogenesis of lymphomas arising in immune-privileged sites appears to be linked with organ-specific immune function and systemic immune activity, the precise biological mechanisms underlying these oncogenic processes remain incompletely understood.

Unlike DLBCL, CLL tends to primarily affect elderly patients, often progresses slowly, and may be asymptomatic during its early stages (13). CLL cases originate from the clonal expansion of abnormal populations of B cells expressing markers such as CD19 and CD20, with these abnormal lymphocyte populations ultimately accumulating in the blood, bone marrow, and lymph nodes. As a class of indolent lymphoma, tumor invasion in CLL cases tends to be relatively limited, and the overall survival of affected patients tends to be relatively long. Even with early treatment, significant improvements in clinical outcomes are rare such that conservative observation is generally adopted in most CLL cases (14). The approach to treating CLL is generally dependent on the stage of disease and overall patient health status and can include observation, immunotherapy, targeted therapy, or immunotherapeutic interventions. As a highly heterogeneous disease, certain patients present with a less aggress form of disease, particularly in cases characterized by a lack of immunoglobulin heavy chain (IGHV) gene mutations, and mutations in the del (17p), del (11q), and TP53 genes (15). CD38 and ZAP-70 are regarded as being indicative of a poor prognosis in immunophenotyping assays (16). Specifically, CD38 is related to IGHV mutational status and may also offer some independent prognostic utility, whereas ZAP-70 is an intracellular protein normally produced in T cells that is commonly dysregulated in CLL cells from certain patients such that it is relevant to patient prognosis (17). CLL progression, in addition to being related to malignant clone characteristics, is also associated with severe immunodeficiency, as these conditions are conducive to the evasion of host immune-mediated detection and elimination. This highlights the closely interrelated, co-evolving nature of tumor cells and their microenvironment over the course of disease progression (18). Monocyte-derived nurse-like cells (NLCs), NK cells, T cells, NKT cells, and mesenchymal stromal cells are all vital environmental components that communicate with CLL cells through a series of chemokine receptors, tumor necrosis factor (TNF) family proteins, adhesion molecules, and soluble mediators (19). CLL can promote the outgrowth of immune cells that suppress immune activity such as regulatory T cells and myeloid-derived suppressor cells, thereby facilitating immune evasion (20). CLL clones often present with characteristics consistent with those of regulatory B cells (Bregs). Indeed, the phenotypes of leukemic B cells and Bregs are often similar, including CD5, CD24, and CD27 expression together with low surface IgM levels. The physiological similarities of these cells also extend to IL-10 production, consistent with their ability to serve as negative regulators of T cell activation and immunity (21). The CD40-CD40L interactions that take place between leukemic B cells and activated CD4+ T cells can promote CLL cell proliferation and anti-apoptotic protein upregulation. T cells can also promote leukemic cell survival through the upregulation of anti-apoptotic Bcl-2 in a process mediated by secreted proteins including IL-4 and IFN-γ (22). Peripheral T cell numbers are raised in patients with CLL, primarily owing to increased CD8+ T cell counts, resulting in decreased CD4:CD8 ratios. As a consequence, even though the overall T cell count is higher, these T cells tend to exhibit reduced functionality including impaired immune synapse formation, limited cytokine production, insufficient degranulation, and decreased cytotoxic antitumor activity (23). These CLL-related T cells also exhibit the overexpression of PD-1 and other marker proteins associated with chronic activation and exhaustion, contributing to the further impairment of their immune synapse formation and cytotoxic activity (24). Key malignant cell features and their disruption of the associated microenvironment additionally contribute to therapeutic resistance in patients. While studies of the CLL-associated microenvironment and BCR signaling have led to the design of targeted therapies including the BTK inhibitor ibrutinib, there are still challenges that remain to be addressed (25). The efficacy of novel immunotherapies has been very limited in CLL as a consequence of the lack of effector T cells in these patients (26). Additional research is thus essential to establish better approaches to treating this form of cancer.

While abnormal B cell growth is a hallmark of both CLL and DLBCL, the two diseases are not causally linked. Indeed, each is characterized by distinct genetic determinants, risk factors, and clinical features. In some instances, patients may nonetheless transition between CLL and DLBCL, as in the rare cases of CLL patients who develop Richter transformation, leading to a more invasive DLBCL diagnosis that tends to progress rapidly while exhibiting biological features and therapeutic responsivity in line with the features of primary DLBCL. The etiological basis for Richter transformation remains incompletely understood, but it is thought to be linked to genetic factors, microenvironmental conditions, and immune escape. Clarifying the differences between these two forms of NHL and the transitions that can arise between the two is vital to optimizing the treatment of affected patients and improving their outcomes.

In this study, a bioinformatics approach was utilized for the identification of key differentially expressed genes (DEGs) between DLBCL and CLL patient samples to assist cancer diagnosis and management. These efforts are valuable, and DEGs can plausibly serve as diagnostic biomarkers suitable for accurately distinguishing between DLBCL and CLL in the clinic in the early stages of diseases, ensuring that clinicians are able to accurately select an appropriate treatment plan given that the two diseases require very different therapeutic approaches. Some of these DEGs may also offer prognostic value, supporting the precise formulation of personalized treatment plans. DEGs are also ideal candidate targets for new or existing therapeutic agents. If a given gene is overexpressed in DLBCL but absent in CLL, for instance, then any drug targeting that gene would exhibit differential efficacy between these two cancers. On the whole, studies of these DEGs will assist the understanding of the mechanisms underlying DLBCL and CLL pathogenesis, benefitting overall research focused on the targeted treatment of these and other diseases.

The implementation of bioinformatics analyses and Raman spectroscopy-based approaches to identifying DEGs through the comparison of DLBCL and CLL samples is thus invaluable as a means of improving diagnostic accuracy for these two forms of disease, supporting personalized treatment, novel drug development, and a greater overall understanding of the mechanistic basis for these diseases.

## Materials and methods

### Data source

Data were downloaded from the Gene Expression Omnibus (GEO) database (http://www.ncbi.nlm.nih.gov/geo/) and included the GSE57083 dataset (species: *Homo sapiens*, 20 samples collected with the GPL570 Affymetrix HG-U133-Plus_2 Array platform) and the GSE68950 dataset (species: *Homo sapiens*, 7 total samples collected with the GPL3921 Affymetrix HG-U133A Array platform).

### Gene expression analyses

Data (CEL format) were analyzed with R (v 3.6.2) to perform the background correction and normalization of these expression data, converting the format, adding missing values, performing background correction with the MAS method, and standardizing data using quantiles. DLBCL data were separated into the DLBCL and CLL groups to screen for DEGs with unpaired t-tests performed with the limma package, using Benjamini and Hochberg (BH)-corrected P values. DEGs were defined by a P < 0.05 and a |logFC| > 1. DEG heatmaps were developed using the pheatmap package in R.

### Functional enrichment

The online DAVID tool (https://david.ncifcrf.gov/) was used for DEG annotation in the GO biological process (GO-BP), cellular component (GO-CC), and molecular function (GO-MF) categories. KEGG pathway enrichment was also performed.

### Protein-protein interaction networks

STRING 11.0 (https://sring-db.org/) was utilized for the construction of the DEG PPI network, followed by the use of Cytoscape to compute the degree centrality scores for the nodes, with higher scores being indicative of more critical nodes. The functions of the 10 top DEGs in the network were further evaluated as potential key nodes.

### Sample collection

**Figure 1:**
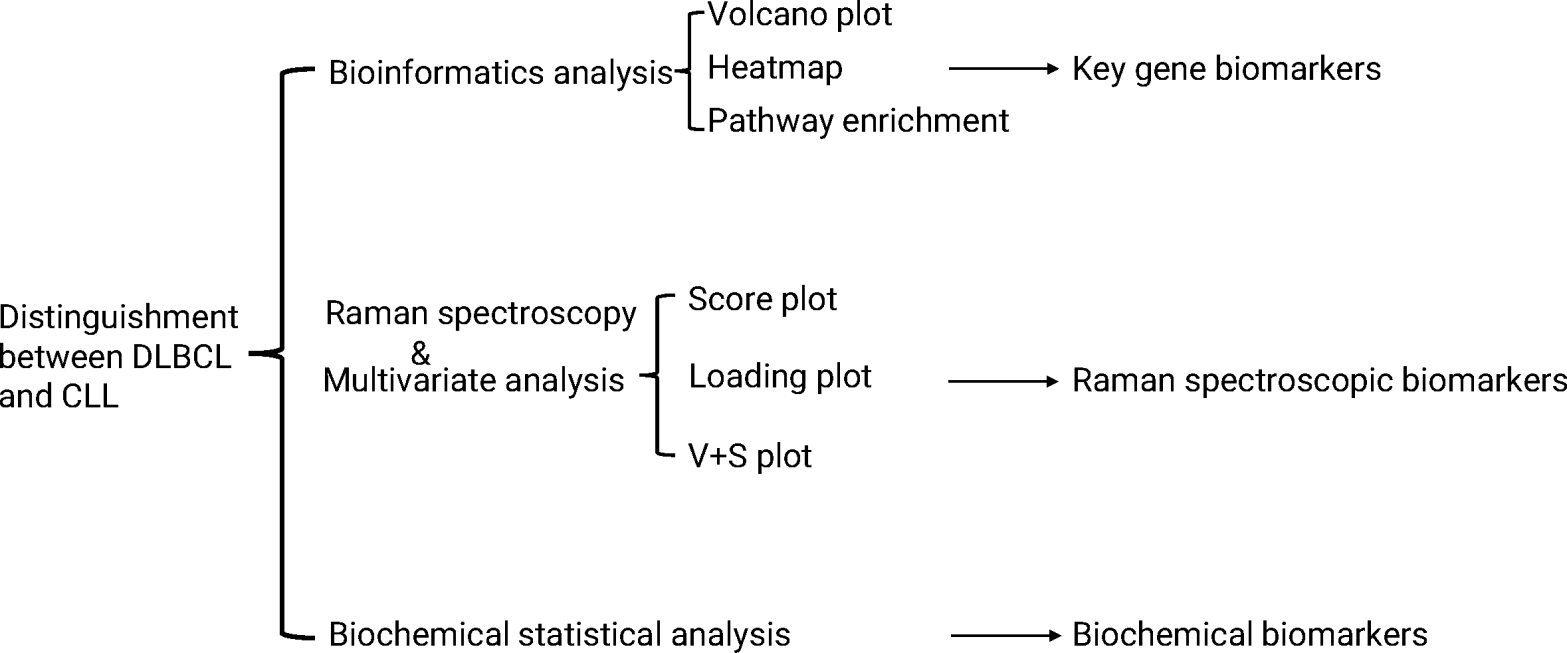
A schematic overview of the Raman spectroscopy and bioinformatics-based analysis of DLBCL and CLL samples. Raman spectra were obtained and analyzed through multivariate statistical analyses performed with SIMCA 14.1.

Serum biochemical analyses were performed with patients recruited from the Hematology Hospital of the Chinese Academy of Medical Sciences (Institute of Hematology, Chinese Academy of Medical Sciences). These 56 patients included 18 DLBCL patients (8 female, 10 male; age range: 25-71 years) and 38 CLL patients (13 female, 25 male; age range: 35-76 years) (**Supplementary Table S1**). Raman spectroscopy, ELISA and Real-time fluorescence quantitative PCR analyses were performed using blood samples from patients who visited the Hematology Hospital of the Chinese Academy of Medical Sciences from January 2024 to April 2024, including 5 men and 4 women between 31 and 73 years. For all patients, bone marrow puncture was used to assess bone marrow cell morphology, conduct flow cytometry analyses, perform electron microscopy, and conduct fusion gene, chromosome, and tissue cell chemistry analyses. Patient clinical diagnoses were based on these results and were confirmed according to FAB. These patients included 4 CLL patients, 5 DLBCL patients, and 5 controls. The Ethics Committee (KT2020016-EC-2) of the Hematology Hospital of the Chinese Academy of Medical Sciences approved this study. Routine serum biochemical testing was performed for all patients in the Clinical Testing Center of the Hematology Hospital of the Chinese Academy of Medical Sciences. A study overview is illustrated in **Figure 1**.

### Clinical data collection

For each participant, medical history, clinical data, and blood-related biomarkers were collected. Briefly, after 10 h of fasting, blood was collected and biochemical indices in the sera were quantified with a fully automated biochemical analyzer. Analyzed parameters included white blood cells (WBC), platelets (PLT), hemoglobin (HGB), neutrophil percentage (NEU%), absolute neutrophil count (NEU #), lymphocyte percentage (LYMPH%), absolute lymphocyte count (LYMPH #), total protein (TP), albumin (ALB), globulin (GLB), alanine transaminase (ALT), aspartate transaminase (AST), alkaline phosphatase (ALP), glutamyl transpeptidase (GGT), total bile acid (TBA), total bilirubin (TBIL), direct bilirubin (DBIL), urea (UREA), creatinine (CREA), uric acid (UA), lactate dehydrogenase (LDH), creatine kinase (CK), CK isoenzyme (CK-MB), 5-hydroxybutyrate dehydrogenase (a-HBDH), amylase (AMY), potassium (K), sodium (Na), chlorine (Cl), calcium (Ca), phosphorus (P), magnesium (Mg), carbon dioxide combining power (CO2CP), glucose (GLU), triglycerides (TG), total cholesterol (TC), high/low-density lipoprotein (HDL and LDL), folic acid (FA), vitamin B12 (B12), ferritin (F), erythropoietin (TPO), iron (Iron), unsaturated iron (UIBC), total iron binding force (TIBC), iron saturation (ISAT), prothrombin time (PT), international standardized ratio (INR), partial thromboplastin time (APTT), thrombin time (TT), fibrinogen (FIB) antithrombin III activity (ATI I), fibrinogen decomposition products (FDB), D-dimer quantification (DD), immunoglobulin G (IgG), immunoglobulin A (IgA), immunoglobulin M (Ig), complement C3 (C3), complement C4 (C4), C-reactive protein (CRP), and rheumatoid factor Anti Streptolysin O (ASO) levels.

### Raman spectroscopy

After adding 5 μL of serum to calcium fluoride or quartz slides, samples were analyzed with a confocal Raman spectrometer XploRA Raman microscope using a 40x objective lens, a 785 nm excitation laser, and a 10 mW output power with the sample fixed on the XYZ 3D platform. Imaging was performed with a 40 0.6 NA Nikon lens, with a spot size range of ~2 × 2 μm exposed to the laser beam. Measurements were taken in the 600-1800 cm^-1^ range, with 6 measurement points per group at a 1 cm^-1^ resolution. Raman spectra for quartz glass slides were analyzed as the background signal. Smoothing, background correction, and baseline correction were performed with the Labspec6 software. Intensity values for all spectra were normalized based on the 1450 cm^-1^ Raman peaks, which were used as internal standards.

### Clinical and Raman spectroscopy data analyses and model development and establishment

OPLS-DA analyses of clinical data from the CLL and DLBCL sample groups and the Raman spectral data from the control, CLL, and DLBCL sample groups were performed with the SIMCA14.1 software. OPLS model performance was assessed based on the R^2^ and Q^2^ parameters. Two hundred resamplings were performed by randomly changing the y matrix. Cluster analyses were conducted and ROC curves were plotted. The Raman peaks that differed significantly in the classification model were considered candidate biomarkers using V+S analysis, selecting those peak positions with variable importance (VIP) > 0.5 and a correlation coefficient (or distance from the center in the V+S plot) within the range of equivalent front positions. The significance of the candidate biomarkers was assessed using P < 0.05. Origin software was utilized for analysis.

### Measurement of serum IL-15 levels by ELISA

Blood samples were collected from normal donors, CLL patients, and DLBCL patients. Sera were obtained after centrifugation at 1000 g for 10 min and the IL-15 concentrations in the samples were measured using a human IL-15 ELISA kit (Abclonal Technology, Woburn, MA, USA). A Spark microplate reader (Tecan, Switzerland) was used to measure absorbance at 450 nm and the IL-15 concentrations in the samples were calculated from a standard curve.

### Real-time fluorescence quantitative PCR

Total RNA was extracted from peripheral blood mononuclear cells using an RNA assay kit and was reverse-transcribed to cDNA. Fluorescence quantitative PCR was used to assess the expression of IL-15 using primers designed and synthesized by Beijing Qingke Biotechnology Co., Ltd. (**Supplementary Table S16**). The 20 μL reaction system was composed according to the instructions of TaKaRa’s fluorescent quantitative reagent. The threshold cycle number (Ct) values of IL15 and GAPDH in the samples were obtained using the sample fitting method and, using GAPDH as the internal reference and the relative quantification (RQ) values, the relative mRNA expression of IL15 and PIK3CG was determined in the tissues. The RQ of the target gene was calculated and statistically analyzed based on RQ=2^- Δ Δ Ct^. The qPCR data was normalized using the 2-ΔΔCt method, setting the control group as the baseline (expression level = 1). The specificity of the PCR products was analyzed using dissolution curves to eliminate the influence of primer dimers and non-specific amplification products. The reaction conditions were pre-denaturation at 90°C for 10 seconds, followed by denaturation at 95°C for 5 seconds, and extension at 60°C for 40 seconds, for a total of 40 cycles. A blank control was used for each PCR reaction. The PCR products were analyzed using 2% agarose gel electrophoresis.

### Statistical analyses

Data were analyzed with SPSS 27.0 and GraphPad Prism 9.0. Spectral data were independent of one another. Normally distributed data are presented as means ± SE (standard error) and were compared via one-way ANOVAs with Bonferroni’s multiple comparisons test when homogenously distributed, whereas they were compared Groups with Welch’s ANOVA and Tamhane’s T2 multiple comparisons test when heterogeneous variance. When non-normally distributed, the results are given as medians (interquartile range) and were compared using Kruskal-Wallist tests and Dunn’s multiple comparisons tests.

Clinical data are represented as described above, respectively comparing continuous data that were normally distributed with and without equal variance using unpaired t-tests and Welch’s unpaired t-test. Non-normally distributed continuous data were compared with Mann-Whitney U tests. Categorical data were compared with chi-square and Fisher’s exact tests. P < 0.05 was considered significant.

## Results

### DEG identification

**Figure 2:**
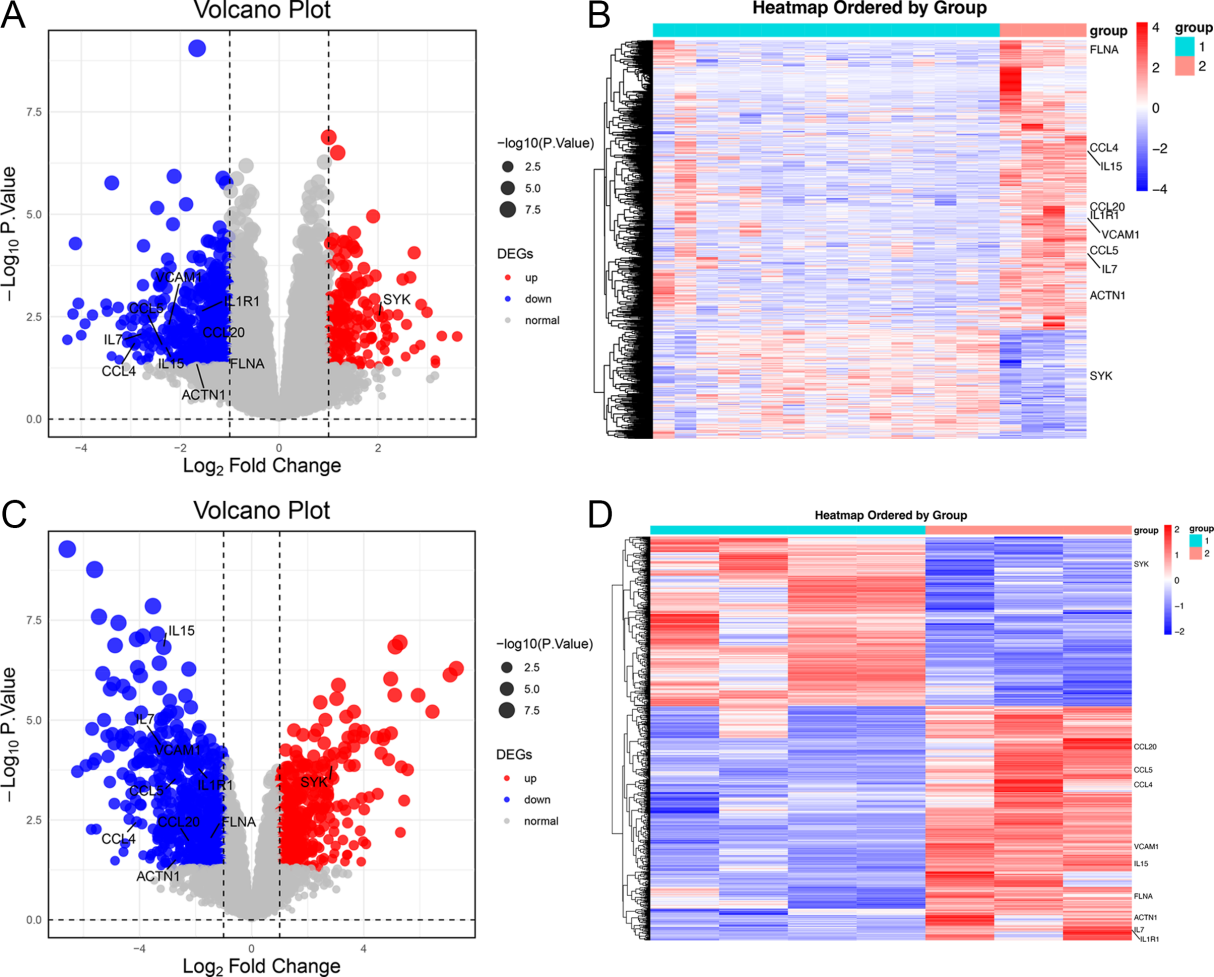
Heatmaps and volcano plots for the GSE57083 and GSE68950 datasets. **(A, C)** Volcano plots for DEGs in GSE57083 **(A)** and GSE68950 **(C)**, with symbols representing individual genes and red or gene respectively denoting up-regulation or down-regulation. P < 0.05 was the significance threshold, and a fold-change value of 1 was established as the threshold for DEG identification. Key genes such as IL15, CCL4, FLNA, CCL5, IL7, VCAM1, ACTN1, CCL20 and IL1R1 were significantly downregulated in DLBCL, indicating their role in modulating immune response and positive regulation of GTPase activity. Key genes such as SYK were significantly upregulated in DLBCL, indicating their role in protein phosphorylation process. **(B, D)** Heatmaps for DEGs in GSE57083 **(B)** and GSE68950 **(D)**, with rows representing genes and columns representing biological samples colored based on grouping.

A total of 715 DEGs (195 upregulated, 520 down-regulated) were identified in the GSE57083 dataset (**Supplementary Tables S4, S5**), whole 1,043 DEGs (438 upregulated, 605 down-regulated) were detected in the GSE68950 dataset (**Supplementary Tables S6, S7**). Of these DEGs, 179 were shared between GSE57083 and GSE68950 (39 upregulated, 140 down-regulated) (**Supplementary Tables S8, S9**). The distributions of these genes were reported using volcano plots (**Figures 2A, C**) and heatmaps (**Figures 2B, D**).

### Functional enrichment analyses

**Figure 3:**
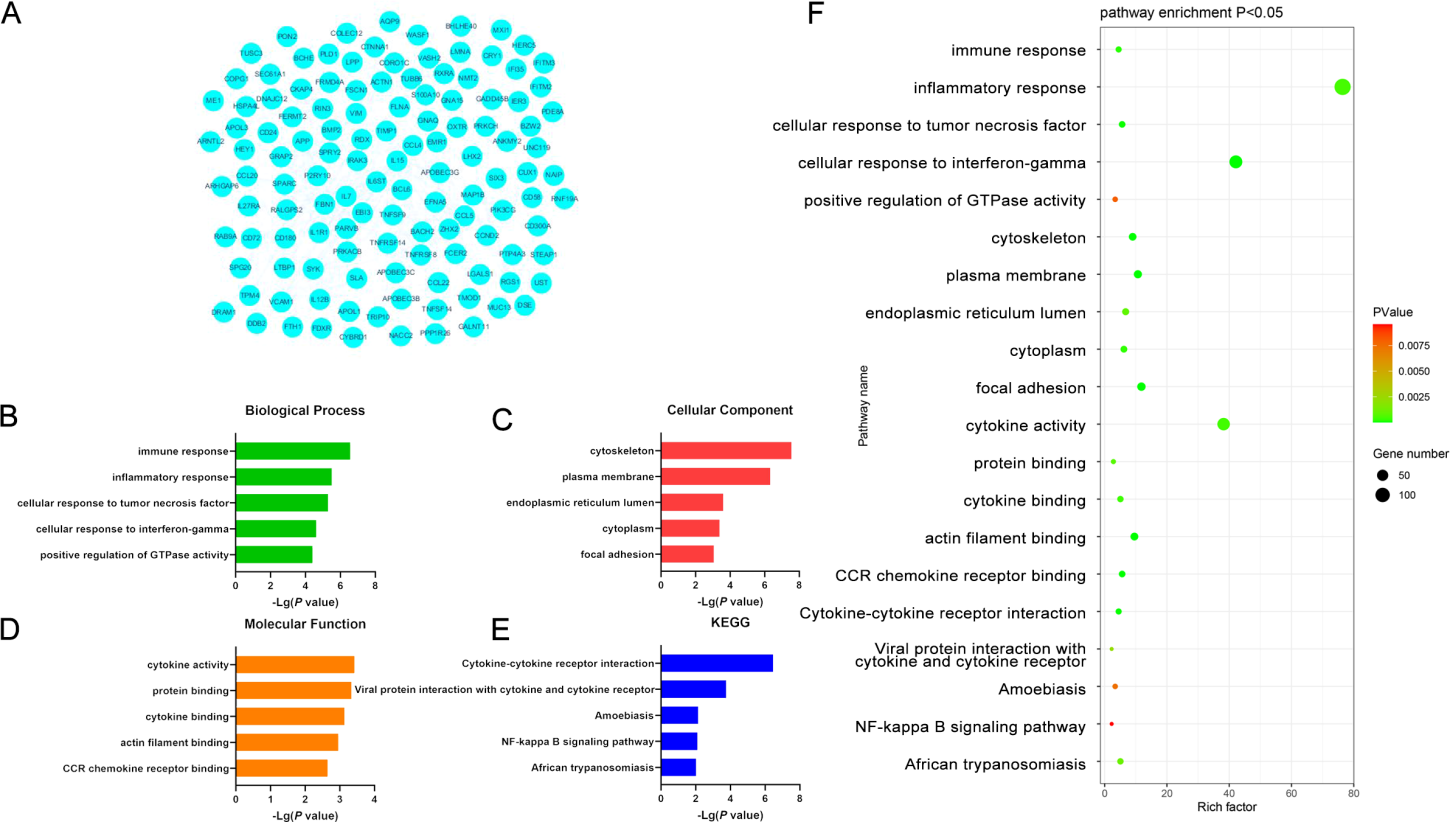
**(A)** Significant DEGs identified when comparing samples from DLBCL and CLL patients. **(B-E)** GO-BP, GO-CC, GO-MF, and KEGG pathway enrichment results for DEGs identified when comparing DLBCL and CLL patient samples. **(F)** Functions and pathways associated with genes most likely to be of value in distinguishing between DLBCL and CLL.

The functions of the overlapping DEGs were examined using the DAVID platform, leading to the identification of 99 enriched GO-BP pathways including the immune response, inflammatory response, cellular response to tumor necrosis factor, cellular response to interference gamma, and positive regulation of GTPase activity pathways. Moreover, DEG enrichment was found in 30 GO-CC terms including the cytoskeleton, plasma membrane, endothelial reticulum lumen, cytochrome, and focal adhesion terms, together with 20 GO-MF pathways of which the most strongly enriched were the cytochrome activity, protein binding, cytochrome binding, actin filament binding, and CCR chemokine receptor binding terms. These DEGs were also enriched in 11 KEGG pathways, the most strongly enriched of which were the Cytokine Cytokine Receptor Interaction, Viral Protein Interaction with Cytokine and Cytokine Receptors, Amoebiasis, NF-κB Signaling, and African Trypanosomatosis pathways (**Figure 3**, **Supplementary Table S10**).

**Figure 4:**
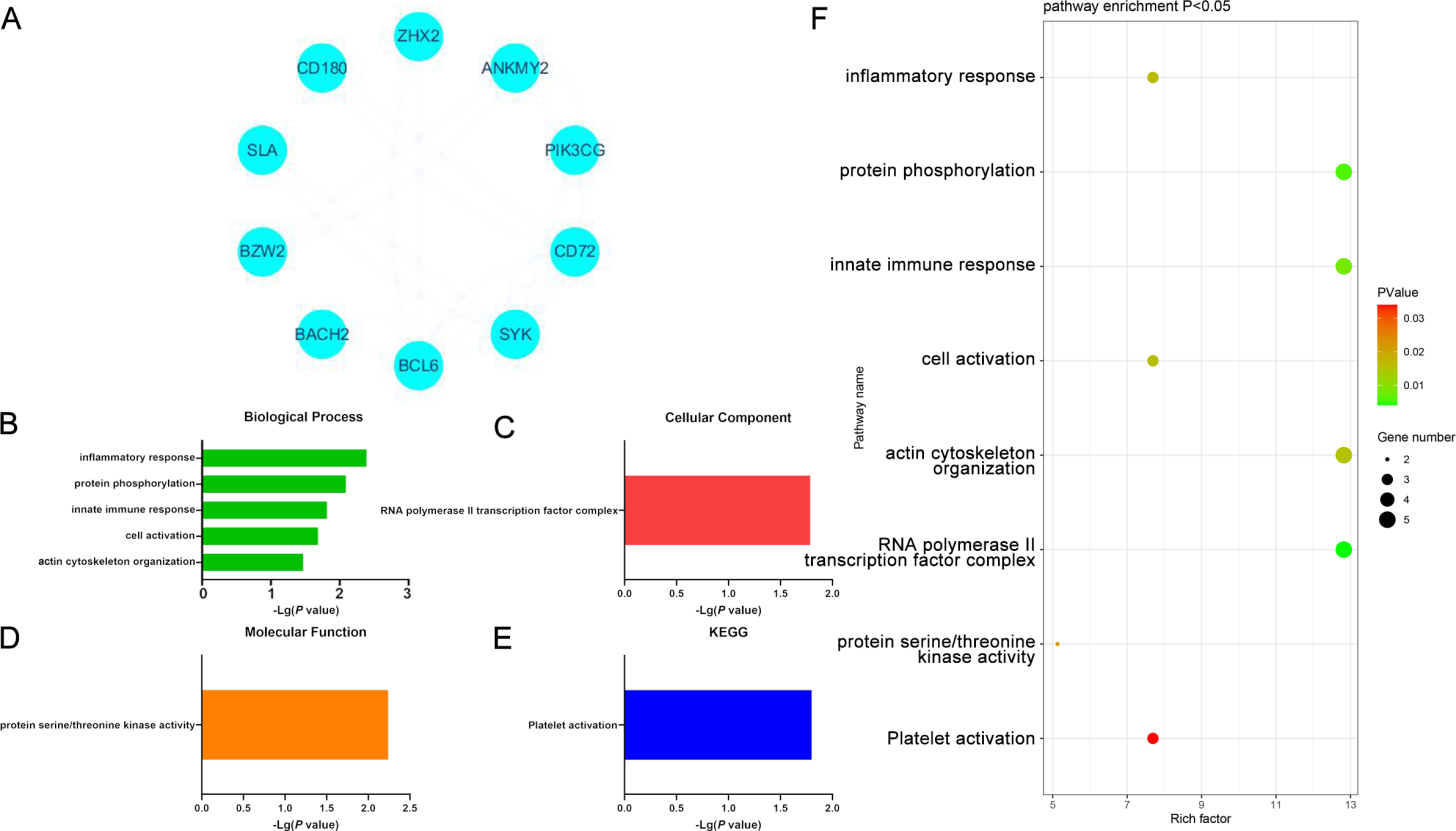
**(A)** Significantly upregulated DEGs identified when comparing samples from DLBCL and CLL patients. **(B-E)** GO-BP, GO-CC, GO-MF, and KEGG results for DEGs identified when comparing DLBCL and CLL patient samples. **(F)** Functions and pathways associated with genes most likely to be of value in distinguishing between DLBCL and CLL.

When specifically focusing on upregulated DEGs, 8 enriched GO-BP terms were detected including the inflammatory response, protein phosphorylation response, innate immune response, cell activation, cytoskeleton, and GTP protein-related response pathways. The only enriched GO-CC term was RNA polymerase II transcription factor complex, while the only enriched GO-MF term was the significant upregulation of protein serine/threonine kinase activity. Protein kinases are key mediators of processes like cell cycle progression, metabolic regulation, and cellular signaling, suggesting that this result may be associated with these functions. These DEGs were also enriched in the platelet activation KEGG pathway, potentially implicating it as a differential mediator of the development of these two forms of cancer. Platelets are closely associated with coagulation, immune activity, and inflammation. These upregulated DEGs may thus play a vital role in driving tumor cell activity in DLBCL (**Figure 4**, **Supplementary Table S11**).

**Figure 5:**
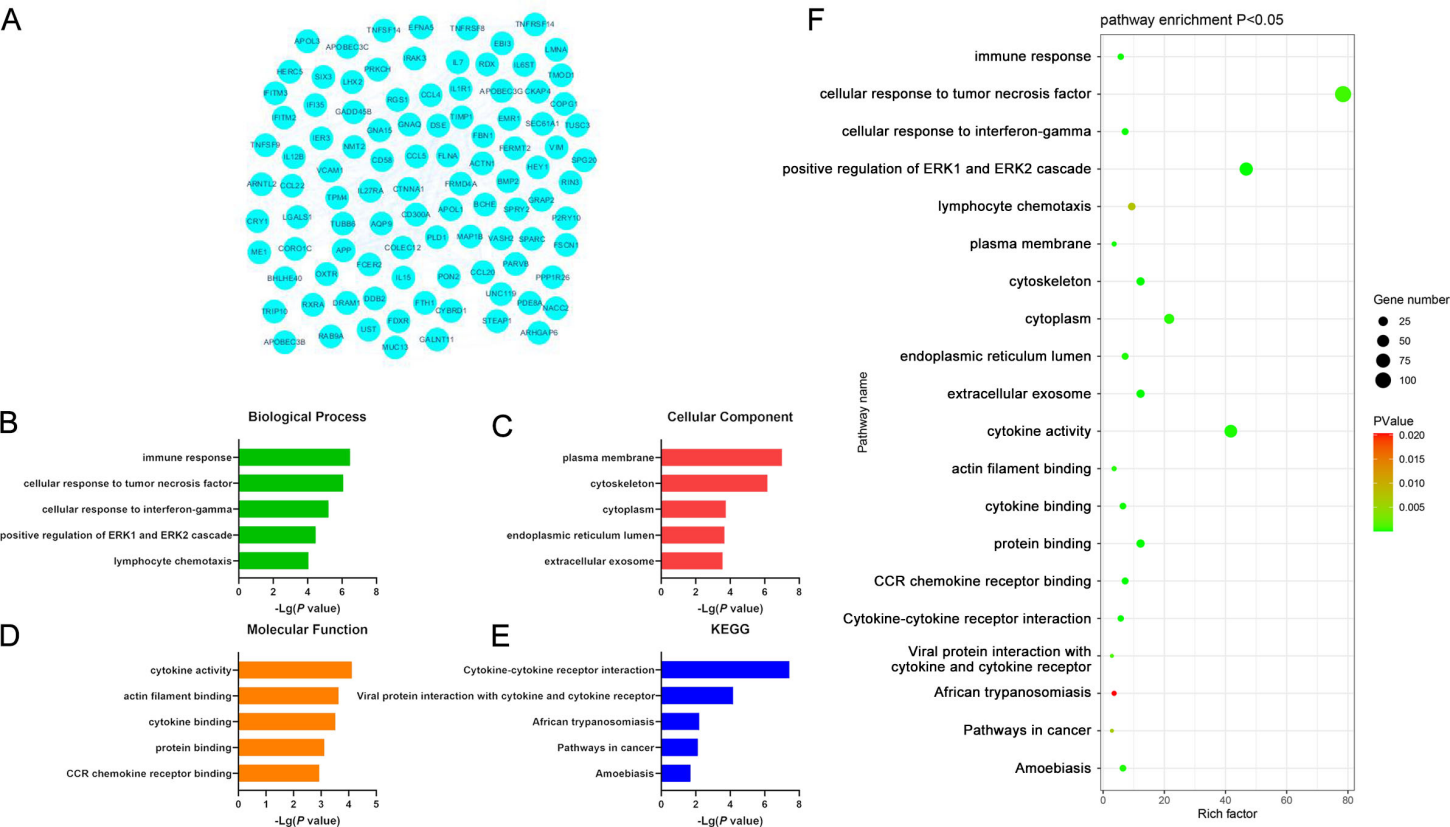
**(A)** Significantly downregulated DEGs identified when comparing samples from DLBCL and CLL patients. **(B-E)** GO-BP, GO-CC, GO-MF, and KEGG results for DEGs identified when comparing DLBCL and CLL patient samples. **(F)** Functions and pathways associated with genes most likely to be of value in distinguishing between DLBCL and CLL.

Similar analyses were also conducted for significantly down-regulated DEGs identified when comparing DLBCL and CLL samples, yielding 90 enriched GO-BP terms including the immune response, cellular response to TNF and IFN-γ, positive regulation of ERK1 and ERK2 signaling pathways, and lymphocyte chemotaxis pathways. Moreover, 30 GO-CC terms were enriched for these DEGs, including the plasma membrane, cytoskeleton, cytoplasmic membrane, endothelial reticulum lumen, and extracellular exosome terms that may play roles related to structural preservation, signal transduction, and material transport. Additionally, 20 enriched GO-MF terms were identified including the cytokine activity, actin filament binding, cytokine binding, protein binding, and CCR chemokine receptor binding pathways potentially associated with cytoskeletal stabilization, the release of cytokines, and interactions between cells. These molecular functions are also relevant to cellular migration, immune activity, and inflammatory response regulation. These downregulated DEGs were also enriched in 9 KEGG pathways including the Cytokine Cytokine Receptor Interaction, Viral Protein Interaction with Cytokine and Cytokine Receptors, African Trypanosomatosis, Pathways in Cancer, and Amoebiasis pathways. These enriched pathways may be related to oncogenic progression, cytokine signaling, viral infections, and the pathogenesis of amoebiasis and other forms of disease. In CLL, the genes enriched in these pathways may thus be closely associated with the emergence of malignant cells, their development, and the modulation of normal physiological activity (**Figure 5**, **Supplementary Table S12**).

### PPI network analyses

**Figure 6:**
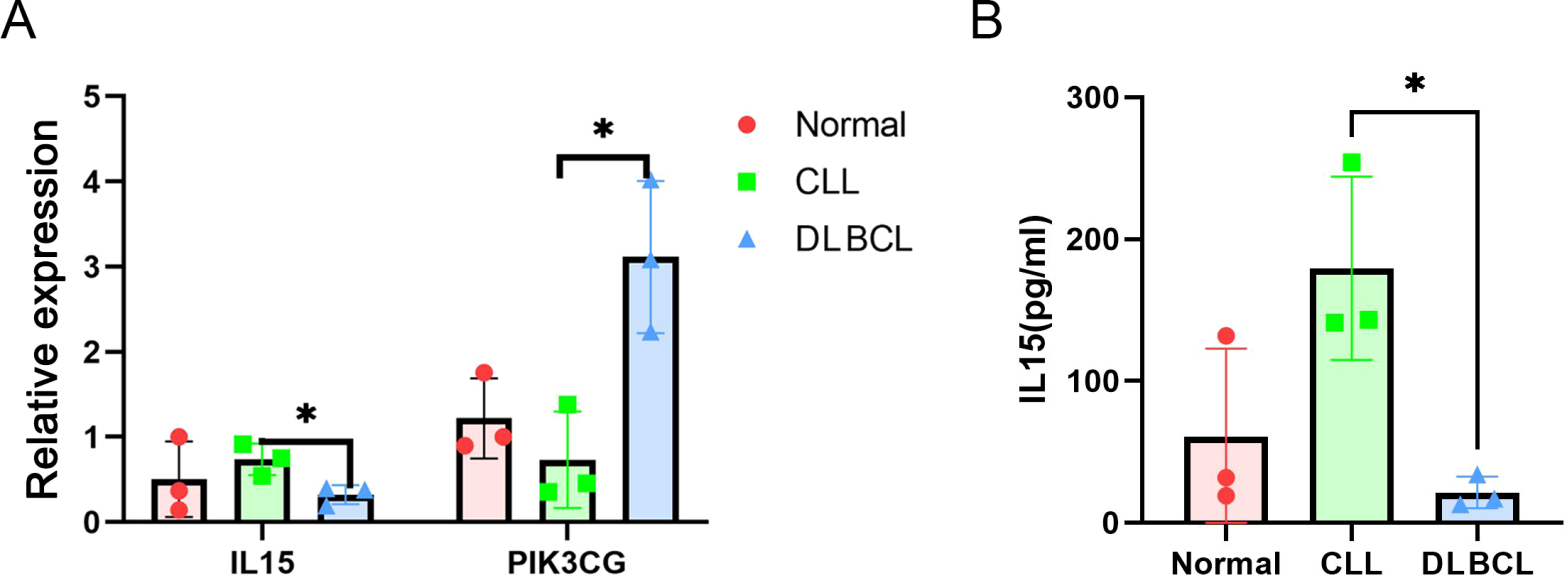
**(A)** Validation of the expression levels of IL15 and PIK3CG using qRT-PCR. **(B)** Measurement of IL15 concentrations in the serum of DLBCL and CLL patients using ELISA. *P ≤ 0.05.

A PPI network was next constructed using the DEGs overlapping between the GSE57083 and GSE68950 datasets incorporating 125 nodes and 418 edges (**Figure 3A**). The network had a high topology score, allowing for the identification of the key nodes within the network. The nodes with the top 10 degree values are presented in **Supplementary Table S13**. After visualizing this PPI network using STRING and the Cytoscape plugin, 10 key DEGs were selected including IL15, CCL4, FLNA, CCL5, IL7, VCAM1, SYK, ACTN1, CCL20, and IL1R1. A majority of these DEGs were classified as down-regulated in the above analyses, indicating markedly higher expression in CLL samples. The PPI network constructed for upregulated DEGs (10 nodes, 20 edges) is presented in **Figure 4A**, and the top 10 key DEGs identified in this network were ANKMY2, PIK3CG, BZW2, BACH2, BCL6, ZHX2, SYK, CD72, CD180, and SLA (**Supplementary Table S14**). The PPI network constructed for downregulated DEGs (100 nodes, 332 edges) is presented in **Figure 5A**, and the top 10 key DEGs identified in this network were IL15, VCAM1, FLNA, CCL5, CCL4, IL7, CCL20, IL1R1, ACTN1, and APP (**Supplementary Table S15**). qRT-PCR was performed to verify the mRNA expression levels of IL15 and PIK3CG. It was found that IL15 was strongly expressed in the peripheral blood of CLL patients, significantly higher than its expression in the peripheral blood of DCBCL patients. In contrast, PIK3CG showed significantly higher expression in the peripheral blood of DCBCL patients than in patients with CLL (**Figure 6A**). The expression levels of both genes were consistent with the bioinformatics results. The IL15 concentrations in the sera of DLBCL and CLL patients were measured by ELISA, showing that its levels were significantly higher in patients with CLL compared with DCBCL patients (**Figure 6B**).

### Establishment of a multi-parameter approach to distinguishing between DLBCL and CLL

**Figure 7:**
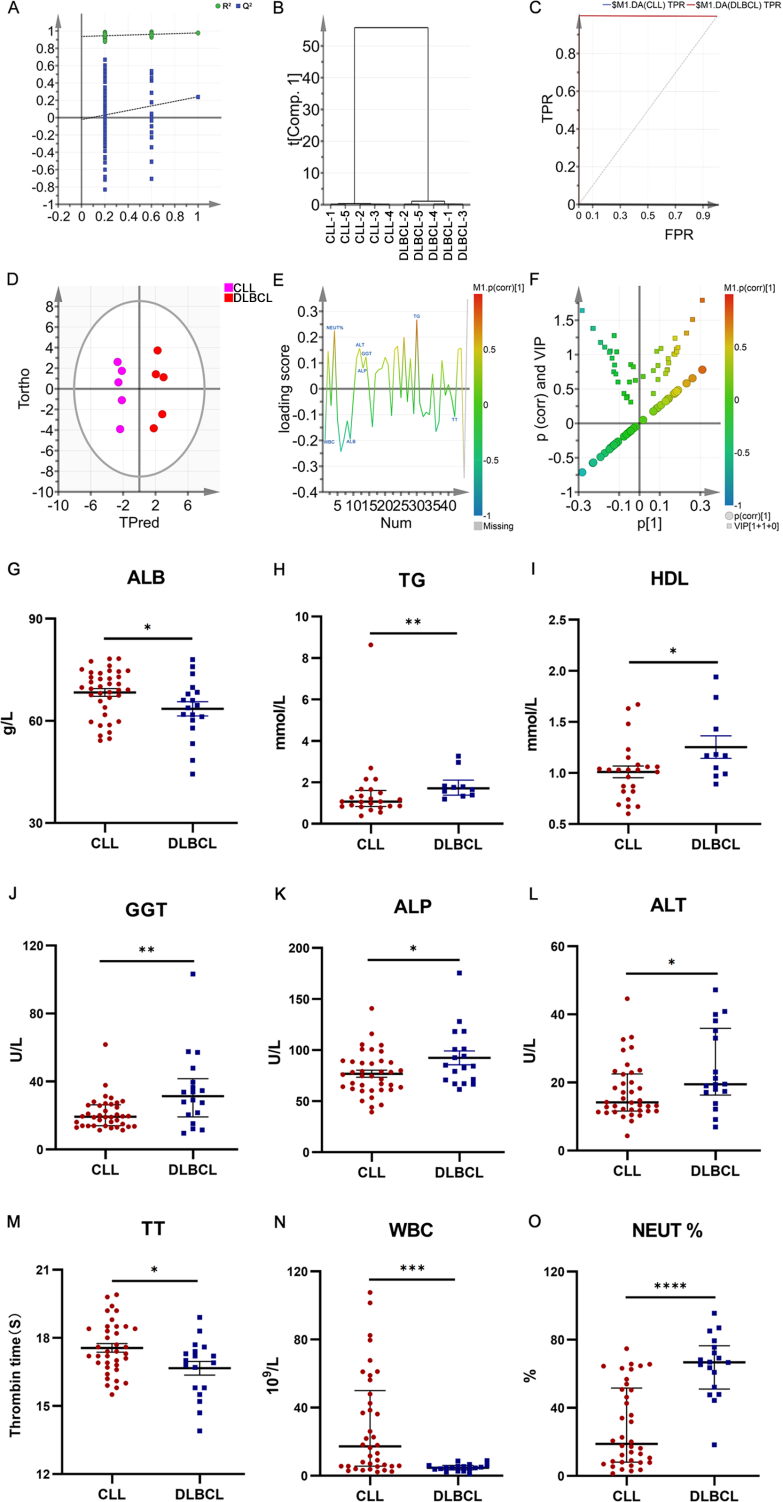
**(A)** OPLS-DA permutation plots for the DLBCL and CLL groups. **(B)** Cluster analysis. **(C)** ROC curves for the DLBCL and CLL groups, both exhibiting AUC values of 1. **(D)** DLBCL and CLL discrimination OPLS-DA score plot showing Hotelling’s 95% confidence ellipse. **(E)** CLL and DLBCL loading plot. **(F)** V+S plot for CLL and DLBCL **(G)** ALB. **(H)** TG **(I)** HDL. **(J)** GGT. **(K)** ALP. **(L)** ALT. **(M)** TT. **(N)** WBC. **(O)** NEUT%. *P ≤ 0.05, **P ≤ 0.01, ***p ≤ 0.001.

Next, a multi-parameter analysis approach was implemented to differentiate between DLBCL and CLL samples by combining these two groups into a single dataset. The SIMCA-P application was then used for supervised orthogonal partial least squares discriminant analysis (OPLS-DA) of the sample data, enabling detailed analyses and comparisons. Permutation plots (**Figure 7A**), clustering analysis plots (**Figure 7B**), and ROC plots (**Figure 7C**) were utilized to assess the discriminatory performance of the supervised OPLS-DA models. Permutation was also used to assess model validity, with permutation analyses revealing that the Q^2^ Y-intercept was negative, consistent with a valid model free of overfitting (**Figure 7A**). Clustering analyses also confirmed that this model exhibits good discriminatory performance on each set of samples. The generated ROC curves yielded AUC values of exactly 1 for both DLBCL and CLL (**Figure 7C**), indicative of high accuracy and the ability to reliably distinguish between these two cancers.

### Potential biochemical biomarker selection and verification

To more fully probe the links between DLBCL and CLL, the OPLS-DA model was further used to screen for potential biomarkers associated with these hematological malignancies. The OPLS-DA score plot (**Figure 7D**) includes a horizontal axis corresponding to score values for the main components during OSC analyses, whereas the vertical axis corresponds to scores for orthogonal components during this analysis. The clear clustering of the two sets of samples in this plot confirmed the successful differentiation between these two malignancies with this model, providing a foundation for efforts to further screen for the biomarkers that can distinguish between the two. When constructing the OPLS-DA loading plot to conduct preliminary screening for the factors that contribute to the effective differentiation between DLBCL and CLL (**Figure 7E**), correlations were noted between the loading and score plots such that factors with higher positive values on the vertical axis of the loading plot tended to exhibit higher positive values on the horizontal axis of the score plot, with the same also being true for negative values. The OPLS-DA VIP plot also exhibited associations between peak VIP values and model correlation coefficients (**Figure 7F**), with redder peak coloration being indicative of a stronger correlation coefficient for that peak position in the discrimination model. A V+S plot was also constructed incorporating VIP and correlation coefficient values in a single figure wherein individual points correspond to a given indicator of interest and the redder the coloration of that point, the stronger the correlation coefficient consistent with a greater contribution to the overall performance of the classification model. In contrast, bluer coloration is indicative of weaker contributions to this classification model. Points situated further from the center of the V+S plot also made greater contributions to the performance of the classification model. This V+S plot was thus used for further biomarker screening, testing for significance based on characteristic peak positions in subsequent analyses as a means of clarifying which features were able to reliably differentiate between DLBCL and CLL (**Figure 7F**). The VIP values for relevant factors in this classification model were used to assess the contributions of these factors to the model, providing a list of hematological indices ranked based on their VIP values, with VIP > 0.5 and biological significance being used to guide biomarker selection. When the peripheral blood biochemical data from study subjects were analyzed (**Figures 7G–O**), ALB, TT, and WBC levels were markedly increased in the CLL group relative to the DLBCL group (P<0.05), whereas the opposite was true for TG, HDL, GGT, ALP, ALT, and NEUT% levels (P<0.05).

### Raman spectroscopy of sera from controls and CLL and DLBCL patients

**Figure 8:**
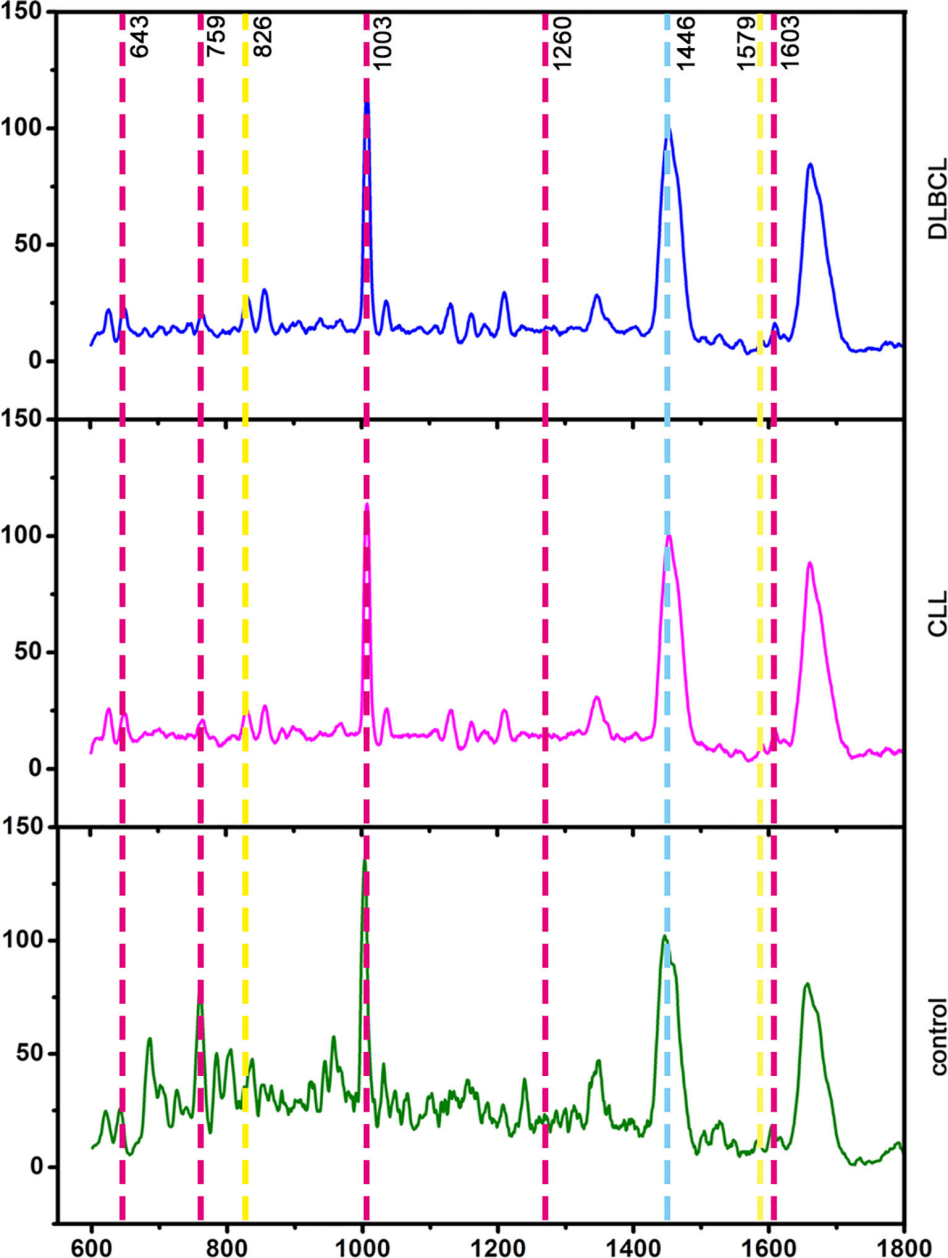
Average serum spectra for the Control, CLL, and DLBCL groups (from bottom to top).

In total, 25, 29, and 30 serum Raman spectra were obtained from the CLL, DLBCL, and control patient samples. The corresponding peak assignments are shown in **Supplementary Table S3**. Raman spectra for these groups were detectable in the 600-1800 cm^-1^ tange and were broadly similar, consistent with the generally comparable composition of the compounds present in samples from these three groups (**Figure 8**). Based on the visual inspection of these spectra alone, differentiating between these three groups is impossible, underscoring the need for a multivariate statistical classification model.

### Development of a Raman spectroscopy-based method to distinguish between CLL, DLBCL, and control samples

**Figure 9:**
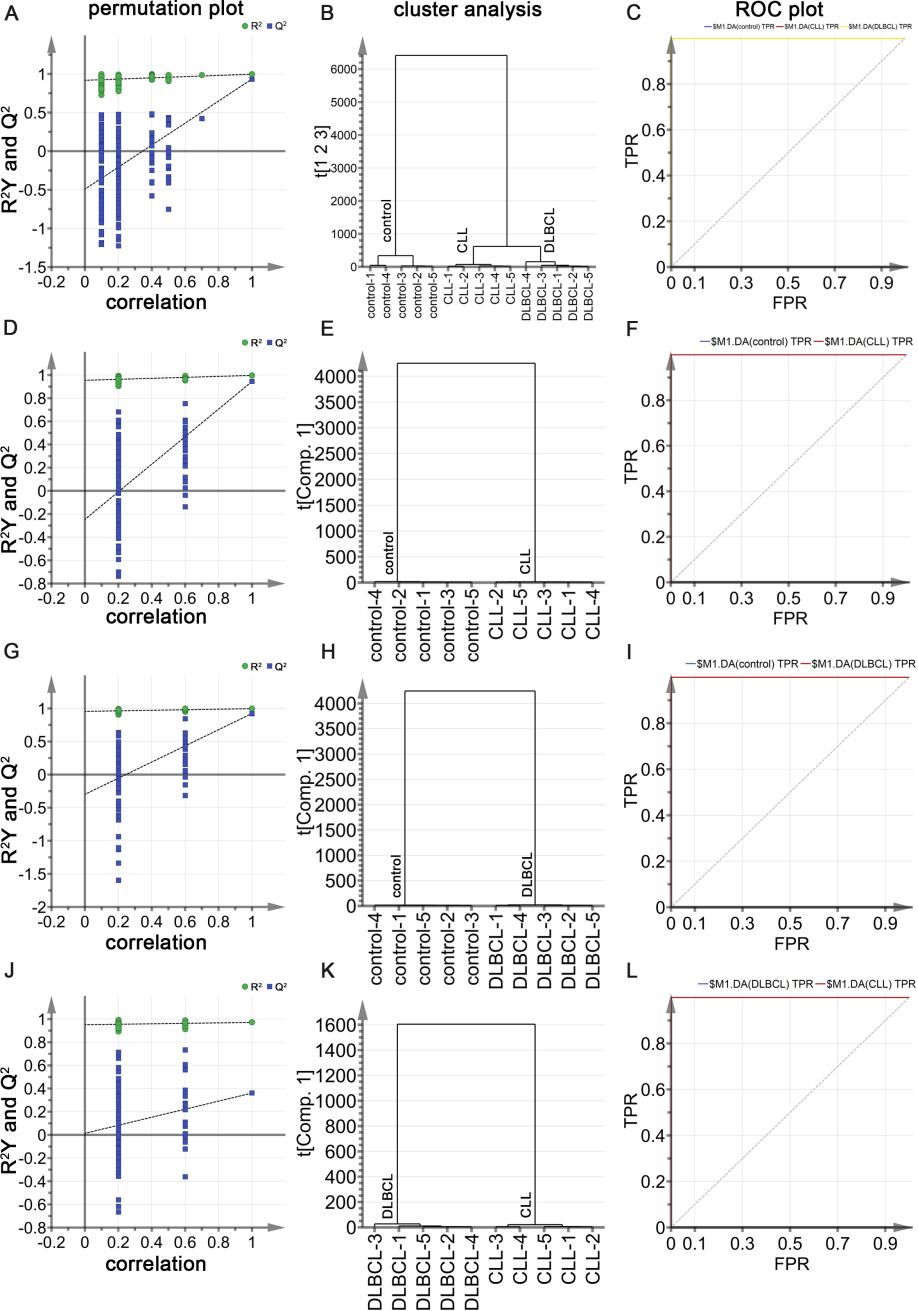
**(A)** OPLS-DA permutation plots for control, CLL, and DLBCL samples. **(B)** Cluster analyses of the groups. **(C)** ROC curves for the groups, each with an AUC value of 1. **(D-F)** Permutation plot **(D)**, cluster analysis **(E)**, and ROC plot **(F)** results for the control and CLL groups, with the latter exhibiting AUC values of 1 for both CLL and control samples. **(G-I)** Permutation plot **(G)**, cluster analysis **(H)**, and ROC plot **(I)** results for the control and DLBCL groups, with the latter exhibiting AUC values of 1 for both DLBCL and control samples. **(K, L)** Permutation plot **(J)**, cluster analysis **(K)**, and ROC plot **(L)** results for the CLL and DLBCL groups, with the latter exhibiting AUC values of 1 for both CLL and DLBCL samples.

Raman spectral data from the control, CLL, and DLBCL patients were next used to conduct supervised OPLS-DA analyses. In the permutation analyses, no overfitting was observed as evidenced by a Q^2^ value with a negative Y-intercept consistent with OPLS-DA model validity (**Figure 9A**). Cluster analyses showed that the OPLS-DA model could differentiate among the three groups with 100% accuracy (**Figure 9B**). The ROC curves for all three sample types using this model were exactly 1 (**Figure 9C**), consistent with a high degree of discriminant performance.

### Potential Raman biomarker selection and identification

**Figure 10:**
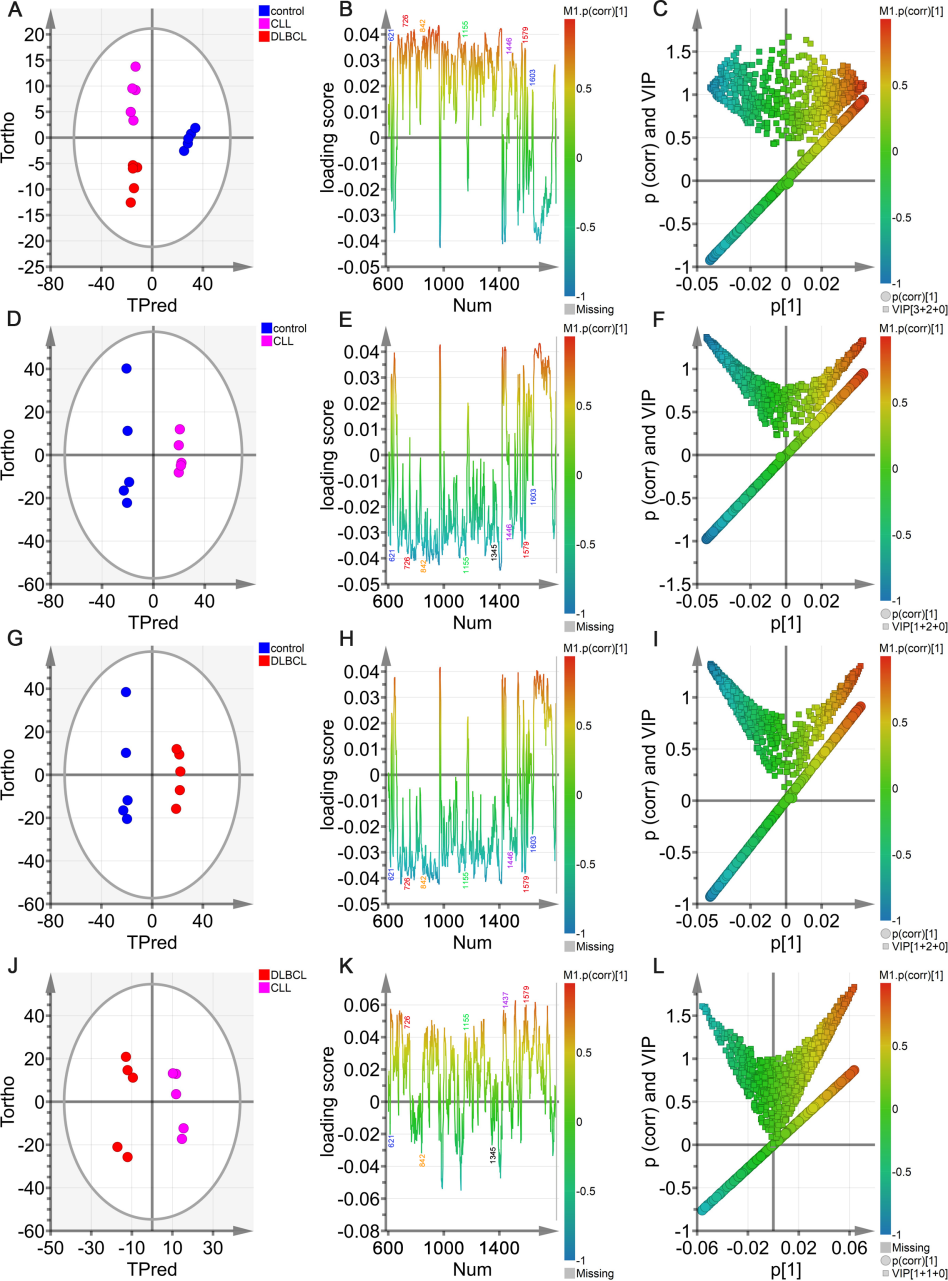
OPLS-DA analyses of sera from the control, DLBCL, and CLL groups. **(A)** Hotelling’s T2 ellipse OPLS-DA score plots showing the 95% confidence zone for the control, CLL, and DLBCL groups. **(B)** Loading plot for the control, CLL, and DLBCL groups. **(C)** V + S plot for the control, CLL, and DLBCL groups. **(D-L)** Hotelling’s T2 ellipse score map **(D, G, J)**, loading plot **(E, H, K)**, and V+S plot **(F, I, L)** for the control and CLL **(D-F)**, control and DLBCL **(G-I)**, and CLL and DLBCL **(J-L)** groups.

To more fully clarify the utility of specific biomarkers capable of distinguishing among control, CLL, and DLBCL serum samples, the OPLS-DA model was further analyzed using an approach similar to that outlined above. In the OPLS-DA score plot (**Figure 10A**), the horizontal axis enabled the visualization of differences among groups whereas the vertical axis enabled differentiation among samples. As all three sample groups were clearly differentiated in the generated plot, with control samples on the positive X-axis whereas the DLBCL and CLL samples were on the negative X-axis, these results confirmed the ability of this OPLS-DA model to effectively distinguish between these different sample types. These results provided a foundation for the further examination of metabolic differences among control, CLL, and DLBCL samples. The OPLS-DA loading plot utilized for preliminary screening of the Raman peaks contributing to the classification model for control, CLL, and DLBCL samples is shown in **Figure 9B**. Peaks in red, blue, green, purple, orange, and black respectively correspond to nucleic acids, proteins, β-carotene, lipids, carbohydrates, and collagen. A correlation was evident between the score plot and the loading plot such that peak positions with larger values on the positive end of the loading plot vertical axis tended to be present at relatively higher levels on the positive horizontal axis of the score plot, while the same was also true for negative values. This approach revealed significantly higher peak intensity values for the peaks corresponding to proteins (621, 1603 cm^-1^), nucleic acids (726, 1579 cm^-1^), β-carotene (1155 cm^-1^), carbohydrates (842 cm^-1^), and lipids (1446 cm^-1^) in control samples relative to those from CLL and DLBCL patients (**Figure 10B**).

In the OPLS-DA V+S plot including VIP values and correlations corresponding to peak positions in the classification model (**Figure 10C**), individual points indicate a specific peak position and the color of these points denotes the strength of the correlation coefficient, with red and blue respectively denoting stronger and weaker contributions to the classification model. Peaks further from the plot center contributed more significantly to the model. To more fully investigate the peak positions contributing to the model, the V+S plot was also utilized to screen for promising biomarkers, performing significance testing on characteristic peak positions to clarify those peaks capable of discriminating among control, CLL, and DLBCL samples (**Figure 10C**). BIP values corresponding to the positions of Raman peaks in this classification model were the primary index used to assess the contribution of peak position to the model, yielding a list of Raman peak positions ranked based on VIP values, retaining those peaks with a VIP > 0.5 and biological significance as candidate biomarkers.

Based on the model developed for the different groups, the control group was combined with the CLL and DLBCL samples for OPLS-DA analysis (**Figures 10D–L**). The OPLS-DA score plots for the respective control vs. CLL, control vs. DLBCL, and CLL vs. DLBCL model comparisons are illustrated in **Figures 10D, G, J**. Clear sample clustering was evident in the resultant scatter plots, consistent with the ability of the OPLS-DA models to effectively extract information capable of differentiating among these groups of serum samples, reliably identifying the samples in each model (**Figures 10D, G, J**). The respective loading plots for the control vs. CLL, control vs. DLBCL, and CLL vs. DLBCL models are presented in **Figures 10E, H, K**. These results demonstrated that the control group was associated with peak positions corresponding to proteins (621, 643, 848, 853, 869, 935, 1003, 1031, 1221, 1230, 1260, 1344, 1443, 1446, 1548, 1579, 1603, and 1647 cm^-1^), nucleic acids (726, 781, 786, 1078, 1190, 1415, 1573, and 1579 cm^-1^), and β-carotene (957, 1155, and 1162 cm^-1^). The peak intensities corresponding to sugars (842 cm^-1^), collagen (1345 cm^-1^), and lipids (957, 1078, 1119, 1285, 1299, 1437, 1443, 1446 cm^-1^) was significantly increased in control samples relative to those from CLL and DLBL patients. The V+S plots for the control vs. CLL, control vs. DLBCL, and CLL vs. DLBC models are respectively presented in **Figures 10F, I, L**. These plots offer a list of Raman peak plots ranked based on VIP values suitable for the identification of candidate biomarkers for these three models (**Figures 10C, F, I, L**). Candidate biomarkers were defined as those peaks with a VIP > 0.5 and biological significance. In the four established models, biomarker selection was based on VIP values, correlation coefficients, loading, and the distance from the center of the V+S plots, enabling the identification of key Raman peak positions related to sample classification. During the course of biomarker validation, nonsignificant Raman peak positions were excluded from further biomarker consideration.

### Raman biomarker verification

**Figure 11:**
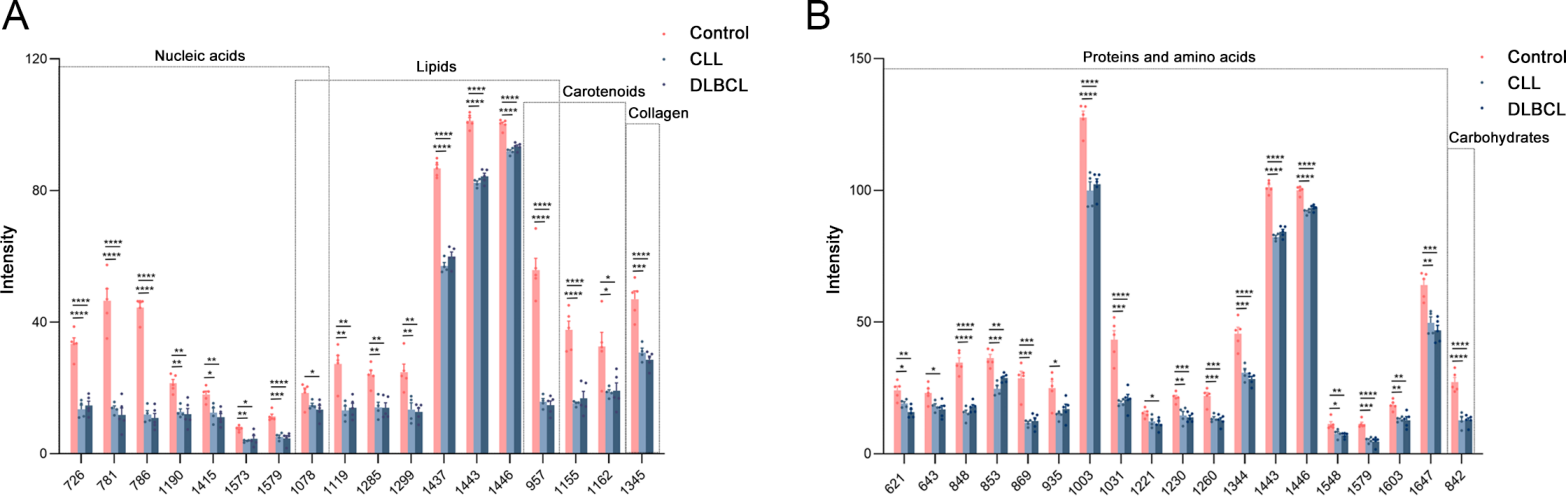
**(A)** Statistical analyses of candidate biomarkers (nucleic acids, lipids, carotenoids, collagen) for the Control vs CLL vs DLBCL comparisons. **(B)** Statistical analyses of candidate biomarkers (proteins, carbohydrates) for the Control vs CLL vs DLBCL comparisons. *P ≤ 0.05, **P ≤ 0.01, ***p ≤ 0.001, ****p = 0.000.

Potential Raman biomarkers were next validated (**Figures 11A, B**), revealing that peak intensities associated with specific proteins in the control group were greater than those in the CLL and DLBCL groups, among which the peaks at 621, 643, 848, 853, 869, 935, 1003, 1031, 1221, 1230, 1260, 1344, 1443, 1446, 1548, 1579, 1603, and 1647 cm^-1^ differed significantly relative to the CLL and DLBCL groups (P<0.05). Similarly, nucleic acid peak intensities in the control samples were markedly greater than those in the CLL and DLBCL groups, with significant differences for the peaks at 726, 781, 786, 1078, 1190, 1415, 1573, and 1579 cm^-1^ (P<0.05). The peak intensities for β-carotene (957, 1155, 1162 cm^-1^) were all significantly higher in control samples relative to CLL and DLBCL samples (P=0.000).

Peak intensity values for carbohydrates (842 cm^-1^) and collagen (1345 cm^-1^) in control samples were significantly greater than those for CLL and DLBCL samples (P<0.001). Similarly, lipid peaks (957, 1078, 1119, 1285, 1299, 1437, 1443, 1446 cm^-1^) exhibited significantly higher intensity values in control samples relative to CLL and DLBCL samples (P<0.05).

To date, there has been no exploration of the utility of Raman spectroscopy as a means of distinguishing between DLBCL and CLL using serum samples, and there is a corresponding lack of information regarding glycolipid metabolism-related serological indices in these patients. This study was thus designed with the goal of developing a model capable of distinguishing among DLBCL, CLL and control samples via Raman spectroscopy combined with multivariate analyses. Screening was also performed for Raman peaks that contributed to the DLBCL and CLL classifications for potential utilization as a biomarker for these cancers, providing a foundation for the effective and rapid detection of early-stage DLBCL and CLL.

## Discussion

DLBCL and CLL are both relatively common hematological malignancies. Intensive research efforts have informed the diagnosis of these cancers, as well as and the creation of targeted therapies, immunotherapies, and allogeneic hematopoietic stem cell transplantation (HSCT) treatment approaches. However, each of these therapeutic strategies has some risk, such as complications, recurrent disease, and death, underscoring a need for further in-depth research exploring the diagnosis, treatment, and prognostic assessment of DLBCL and CLL.

In this study, two independent datasets from the GEO dataset were normalized and analyzed, identifying 179 DEGs when comparing DBLCL and CLL samples, of which 39 and 140 were respectively upregulated and down-regulated. In functional analyses, these overlapping DEGs were enriched in pathways, including immune and inflammatory responses, cellular TNF and IFN-γ responses, and positive regulation of GTPase activities. A core set of 10 key DEGs was selected through further bioinformatics analyses that included IL15, CCL4, FLNA, CCL5, IL7, VCAM1, SYK, ACTN1, CCL20, and IL1R1, with a majority of these genes being significantly overexpressed in CLL relative to DLBCL. To further explore the signaling pathways relevant to the treatment of these genes, DEGs were further screened to identify the top 10 key upregulated DEGs (ANKMY2, PIK3CG, BZW2, BACH2, BCL6, ZHX2, SYK, CD72, CD180, and SLA) as well as the top 10 key down-regulated DEGs (IL15, VCAM1, FLNA, CCL5, CCL4, IL7, CCL20, IL1R1, ACTN1, and APP). The top 5 DEGs in each category are the focus of the remainder of this discussion section.

Vascular cell adhesion molecule-1 (VCAM-1) is an immunoglobulin (Ig) superfamily member and type I transmembrane glycoprotein with multiple Ig-like domains containing disulfide-linked loops (27). VCAM-1 plays a role in the inflammatory process, facilitating the interstitial adhesion of leukocytes and the migration of these cells. Endothelial exposure to inflammatory cytokines can elicit the release of soluble VCAM-1 (sVCAM-1), promoting monocyte, lymphocyte, and eosinophil recruitment, migration, and adhesion (28). VCAM-1 expression is evident in the heart, brain, kidneys, placenta, bladder, lymph nodes, and spleen. The sVCAM-1 levels in patients with atrial fibrillation, ischemic cardiomyopathy, coronary artery disease, and acute myocarditis are elevated, and both VCAM-1 and sVCAM-1 are regarded as biomarkers of autoimmune myocarditis, cancer, and immunological diseases (29–31). Under conditions of persistent inflammation, T cells, macrophages, and/or NK cells can release inflammatory mediators, including IL-1β and TNF-α (32), the latter of which promotes VCAM-1 upregulation and interacts with the receptor TNFR1 to recruit a complex consisting of TRAF2, RIP1, and cIAP1/2 that promotes TAK1 and IKK signaling pathway activity. IKK, in turn, promotes the degradation of the NF-κB inhibitor IκB, thereby driving NF-κB activation and VCAM-1 upregulation (33). miR-126 can promote angiogenic activity while reducing vascular inflammation, inhibiting the extravasation of leukocytes through reductions in VCAM-1 expression. When VCAM-1 is expressed on the endothelial surface, it can interact with the α1 β4 integrins on circulating leukocytes, thereby activating intracellular signaling that disrupts endothelial cell-cell connections and promotes actin remodeling, enabling these leukocytes to extravasate from systemic circulation into the damaged site (34).

PI3K catalytic subunit gamma (PIK3CG) is a class I catalytic PI3K subunit that, like other subunits in this class (p110-α, p110-β, and p110-δ), is capable of binding to the regulatory p85 subunit in the PI3K complex. PIK3CG is involved in the control of cellular activities through the phosphorylation of proteins and lipids (35). PIK3CG can be regulated directly by Ras and G β - γ and Ras in the G protein-coupled receptor (GPCR) pathway, controlling a diverse array of immune- and inflammation-related activities. PIK3CG has also been established as a candidate target for managing certain cancers, including Kaposi’s sarcoma, medulloblastoma, and ALL (36). CLBC cell invasivity, migration, and stem cell maintenance are impaired upon the inhibition of PIK3CG activity (37). Jun et al. have also demonstrated that targeting PIK3CG with PTX can significantly influence CLBC treatment, reducing the risk of recurrent disease by 40% (38). Chung et al. further determined that PIK3CG is an essential regulator of prostate cancer activity related to KRAS activation and p53 deficiency. It may be a viable target in patients with advanced metastatic disease (39). Zhang et al. noted that PIK3CG is targeted directly by RBPJκ-dependent Notch signaling, and the inhibition of PIK3CG in CLBC cell lines can limit the formation of tumor balls and the migration of tumor cells such that it is a promising candidate target for CLBC subtypes.

The transcription factor basic leucine zipper and W2 domains 2 (BZW2), sometimes referred to as eIF5 mimic protein (5MP) 2, is a cytosolic member of the alkaline region leucine zipper (bZIP) superfamily that binds to cadherin and is linked to nervous system development and cellular differentiation. BZW2 is linked with various forms of cancer. For example, Wang et al. demonstrated that TEAD4 upregulation in nasopharyngeal carcinoma, upregulated TEAD4 promotes oncogenic signaling via the AKT pathway through the activation of BZW2 transcriptional activity, driving cancer progression (40). Gao et al. (41) determined that higher levels of BZW2 expression were evident in muscle-invasive bladder cancer tissues relative to normal tissues. The knockout of BZW2 in bladder cancer cells blocked cell cycle progression and apoptosis induction. In fibrosarcoma, BZW2 can promote tumor growth, and it is also expressed at high levels in colorectal and liver cancers (42–48). The dysregulation of BZW2 may thus be a hallmark of certain forms of cancer (49). The oncogenic properties of BZW2 are associated with the translation of ATF4, another transcription factor, delaying the restart and thus promoting the survival of stress-exposed tumor cells. Li et al. conducted a systematic analysis of BZW2 in several HCC-related datasets. They documented a close link between this transcription factor and HCC patient prognostic outcomes, with BZW2 regulating eIF factors via c-Myc signaling (50).

Myc is associated with many important biological pathways. These include: (1) Metabolic pathways, where Myc is a key regulatory factor involved in metabolic reprogramming; (2) Pathways associated with the cell cycle and proliferation, where Myc promotes cell proliferation and modulates cell cycle dynamics. High levels of Myc expression induce replication pressure and genomic instability, sensitizing cells to apoptosis. Cell proliferation requires a doubling of energy and cell biomass, and replicating cells are thus particularly sensitive to deficiencies in oxidative phosphorylation (OXPHOS), and OXPHOS-deficient replicating cells are especially vulnerable to high levels of Myc, as MYC helps them evade metabolic checkpoints and accelerate cell cycle progression; (3) Pathways related to mitochondrial diseases, as upregulation of Myc and/or its typical transcriptional features have been observed in several cellular and mouse models of mitochondrial diseases. Changes in gene expression and metabolite levels associated with the mitochondrial integrated stress response (mt ISR) overlap significantly with Myc overexpression; (4) Pathways associated with the tumor microenvironment, in which Myc interacts with the tumor microenvironment through signaling pathways and molecular networks to modulate processes such as proliferation, replication pressure, and DNA repair in tumor cells, driving tumor development and drug resistance; (5) Transcriptional regulatory pathways, in which Myc acts as a global transcriptional amplifier, binding and increasing the expression of active promoters and controlling the expression of at least 15% of human genes, including genes related to cell cycle progression, metabolism, ribosome biogenesis, and translation.

The transcriptional repressor BTB domain and CNC homolog 2 (BACH2) belong to the BACH family of alkaline leucine zipper transcription factors that are found in the nucleus and cytosol and exhibit the ability to bind DNA in a sequence-specific manner (51). NK cells are key mediators of immune surveillance, protecting against cancer and infection. BACH2 is a vital mediator of adaptive immunity, but how it functions in NK cells and other innate cell populations is poorly characterized. Imianowski et al. found that BACH2 may be a negative regulator of NK cell differentiation, maturation, and function (52). BACH2 can limit NK cell maturation under conditions of weak stimulation, maintaining numbers of undifferentiated NK cells such that knocking out BACH2 in NK cells can lead to cytotoxic NK cell accumulation within tissues, more effectively protecting against lung cancer metastasis. These results support the role of BACH2 as a global negative regulator of cytotoxic activity and NK cell-mediated tumor surveillance.

BCL6 is a transcriptional repressor involved in germinal center (GC) B cell responses, preventing premature GC B cell activation and differentiation and allowing cells to tolerate better the breakage of the DNA that occurs during Ig gene remodeling. The essential role BCL6 plays in B cell development can be coopted by malignant cells such that it serves as an oncogene in GC-derived lymphomas, with high mutation rates and translocation in DLBCL (53). BCL6 functions by binding to target DNA sequences and recruiting co-repressor complexes to suppress transcription. Specifically, its N-terminal BTB domain is responsible for recruiting the BCOR, NCOR, and SMRT co-repressor proteins to the extended groove motif of the BTB dimer interface. At the same time, its core RD2 region can inhibit gene transcription through interactions with MTA2, HDAC2, NuRD, and CBP (54). BCL6 is reportedly upregulated in glioblastoma and gastric, ovarian, and non-small cell lung cancers, and its expression is associated with malignant features, including proliferation, invasivity, migratory activity, survival, and therapeutic resistance (55).

IL-15 is a 14-15 kDa cytokine essential for NK, NKT, and memory CD8+ T cell dynamics. While IL-15 is secreted at very low levels, it can be readily delivered in trans through the unique IL-15r alpha receptor on the surfaces of cells that produce IL-15 such that it can interact with a target cell receptor consisting of IL-2Rβ and common gamma chains. IL-15 and IL-15Rα binding in solution results in the formation of the highly potent IL-15 super antagonist (IL-15 SA) complex, which can robustly activate cells responsive to IL-15, particularly NK cells, promoting a range of antitumor and antiviral effects (56). IL-15 is vital for NK and CD8+ T cell expansion and functionality (57, 58). IL-15 inhibits HIV-specific CD8+ T cell apoptosis and increases these cells’ activation, expansion, cytotoxicity, and IFN-γ production (59). Chehimi et al. showed that IL-15 therapy improved the cytotoxic functions of HIV patient-derived NK cells while significantly reducing apoptotic PBMC death (60). *In vitro*, NK cells can suppress HIV replication, much like CD8+T cells, through a mechanism potentially linked to CC chemokine secretion (61). Further studies of the potential therapeutic capability of IL-15 to remediate NK and CD8+ T cell responses in the context of HIV infection are thus warranted.

Filamin A (FLNA) possesses an N-terminal actin-binding domain (ABD) composed of two calponin homologous CH1 and CH2 domains, two rod-like domains, and 24 Ig repeat sequences that confer actin-binding activity (62). FLNA can crosslink actin filaments, linking them to membrane glycoproteins and serving as a scaffold for signaling intermediates that are involved in cytoskeletal remodeling necessary for interactions with transmembrane receptors, integrins, and second messengers, ultimately shaping cellular shape, migratory activity, motility, and signal transduction (62). Periventricular nodular ectopia (PVNH) is the most commonly associated disease, and it is characterized by impaired neuronal migration such that ectopic neurons accumulate at ventricle edges (63, 64). CCL5 is a C-C motif chemokine family member closely associated with inflammatory and immune regulation. It can induce peripheral monocyte, memory T helper cell, and eosinophil responses, triggering activation and histamine release (65). CCL5 expression is observed in T cells, platelets, macrophages, renal tubular epithelial cells, synovial fibroblasts, and tumor cells (66). CCL5 signals through its cognate receptor CCR5 to promote tumor growth, tumor progression, tumor cell migration and metastasis (67).

In scientific research, especially in the field of biomarker research, the establishment of control groups is crucial to ensure the validity and reliability of the research results. The reasons for including a control group in the selection and identification processes of Raman biomarkers primarily include: (1) The provision of a benchmark, where the control group serves as a reference standard to assist in the confirmation of whether the observed changes represent a true association with the disease under investigation. Without this comparison, it would be difficult to determine whether the observed changes are caused by the disease itself or other factors; (2) Exclusion of bias, as factors other than the disease, such as age, sex, and lifestyle habits, may affect the expression of biomarkers. The control group helps to control these variables, ensuring the accuracy and reproducibility of the results; (3) Statistical efficacy, as statistical analysis relies on the presence of a control group to evaluate whether the observed differences are statistically significant, rather than accidental events. The most significantly enriched pathways for the DEGs between CLL and DLBCL patients were found to be associated with the immune response, cytoskeleton, cytokine activity, and cytokine-cytokine receptor interactions. The genes most significantly associated with these pathways included VCAM-1, ANKMY2, PIK3CG, BZW2, BACH2, BCL6, IL-15, FLNA, and CCL5.

After visualization of the PPI network using the STRING database and Cytoscape plugin, we identified 10 key differentially expressed genes, including IL15, CCL4, FLNA, CCL5, IL7, VCAM1, SYK, ACTN1, CCL20, and IL1R1. Most of these genes were found to be downregulated, indicating that their expression was significantly higher in CLL samples. A PPI network (consisting of 10 nodes and 20 edges) was constructed for the upregulated differentially expressed genes. The top 10 key genes identified in this network were ANKMY2, PIK3CG, BZW2, BACH2, BCL6, ZHX2, SYK, CD72, CD180, and SLA. The protein encoded by PIK3CG is one of the catalytic subunits of phosphatidylinositol 3-kinase (PI3K), and the PI3K signaling pathway plays a crucial role in various biological processes such as cell growth, proliferation, survival, metabolism, and migration. In both DLBCL and CLL, upregulation of PIK3CG may lead to abnormal activation of the PI3K signaling pathway, promoting cancer cell proliferation and survival and inhibiting cell apoptosis, and thus promoting disease progression. For example, overactivation of PI3K signaling may influence downstream target proteins such as Akt, affecting cell metabolism and survival signals, and enabling cancer cells to evade normal cell death mechanisms and continue to proliferate and form tumors. The PPI network constructed for the downregulated DEGs (including 100 nodes and 332 edges) identified the top 10 key genes as IL15, VCAM1, FLNA, CCL5, CCL4, IL7, CCL20, IL1R1, ACTN1, and APP. Among them, IL15 is a multifunctional cytokine that plays an important role in the immune system. In CLL, overexpression of IL15 may promote disease progression through multiple pathways. In terms of promoting the proliferation of cancer cells, IL15 binds to receptors on the cell surface, leading to the activation of a series of intracellular signaling pathways and resulting in the proliferation of leukemia cells. For example, it may activate the JAK/STAT signaling pathway, regulate the expression of cell cycle-related proteins, and drive cells into the proliferation cycle. In terms of apoptosis inhibition, IL15 may also prevent apoptosis in leukemia cells by upregulating the expression of anti-apoptotic proteins or inhibiting the activity of pro-apoptotic proteins, allowing cancer cells to continue to survive and accumulate in the body. It can also affect the tumor immune microenvironment, as IL15 may regulate the functions and activities of immune cells, inhibiting the body’s antitumor immune response. For example, it may affect the differentiation, proliferation, and killing activity of immune cells such as T and NK cells, making it difficult for the immune system to effectively clear leukemia cells.

In an article entitled “Immunophenotypic and genomic landscape of Richter transformation diffuse large B-cell lymphoma,” Nadeu et al. explored the genetic map of Richter’s Transformation, suggesting that diffuse large B-cell lymphoma (DLBCL) represents a more aggressive form of CLL and providing insights into the pathogenesis and treatment of DLBCL. Nadeu et al. mentioned ATM in their most recent article published in 2024, in which BCL2, BIRC3, BTK, CXCR4, EGR2, FBXW7, KRAS, MYD88, NFKBIE, PLCG2, POT1, SF3B1 and a series of genes were analyzed for mutations. Although no significant differences were found in the expression of all these genes, many were found to be differentially expressed in the present study. Nadeu et al. also described multiple genes associated with B-cell pre lymphocytic leukemia (B-PLL) in their article “Epigenic features support the diagnosis of B-cell prolymphatic leukemia and identify 2 clinicobiological subtypes”, mainly including MYC, SF3B1, TP53, CCND1, CCND2, and CCND3. Among them, mutations in SF3B1 are associated with poor prognosis in CLL. The genes mentioned above also overlap with the DEGs in this article, indicating the credibility of our research results (68–70).

Many of the DEGs identified here were related to lipid metabolism, including CCL22, BMP2, CCL20, CCL5, and ACKR3. Among them, the role of CCL20 in lipid metabolism may be related to its function in immune cell chemotaxis and the inflammatory response. CCL20 is associated with lipid droplet accumulation-mediated macrophage survival and regulatory T cell (Treg) recruitment, indicating its potential role in lipid metabolism in the tumor microenvironment. The role of CCL5 in lipid metabolism may be related to its function in immune cell chemotaxis and the inflammatory response. Several studies have found that the expression of CCL5 is associated with the degree of T cell infiltration within tumors, indicating its potential role in lipid metabolism in the tumor microenvironment. ACKR3, the atypical chemokine receptor 3, although its primary function is that of a scavenger receptor for chemokines, plays a role in the negative regulation of cell proliferation and the positive regulation of the ERK1/2 cascade, indirectly affecting lipid metabolism.

The Richter transformation is the process by which chronic lymphocytic leukemia (CLL) transforms into more aggressive lymphomas such as diffuse large B-cell lymphoma (DLBCL). In Iyer’s articles, multiple genes and their roles in CLL and the Richter transformation are mentioned. The genes include TCL1, SF3B1, TP53, CDKN2A/CDKN2B, MYC, and NOTCH1. Abnormalities in the TCL1 gene are common in CLL patients and its overexpression is associated with the clonal expansion of B cells. SF3B1 mutations are commonly seen driver mutations in CLL, and the gene is involved in RNA splicing processes. Its mutation is related to the progression and transformation of CLL. Mutations in the TP53 gene are associated with high-risk subtypes of CLL, and TP53 mutations are typically associated with the Richter transformation, leading to disease progression. The deletion of the CDKN2A/CDKN2B genes is associated with the progression of CLL and the Richter transformation. Their absence may lead to abnormal cell cycle regulation, thereby promoting tumor development. Mutations and deletions of these genes play important roles in the occurrence, development, and response to treatment of CLL and the Richter transformation. Understanding the functions and interactions of these genes is thus crucial for developing new therapeutic strategies (71, 72).

The present study identified key genes and pathways associated with these two diseases through bioinformatics analysis followed by experimental verification. These analyses revealed many upregulated and downregulated DEGs, and determined through functional enrichment analysis that these genes are closely related to inflammation and the immune response. In addition, the author also analyzed serum samples from patients using Raman spectroscopy and found significant differences in spectral characteristics between the control, CLL, and DLBCL groups. These spectral data were combined with the DEG data to successfully distinguish between these two diseases using the supervised orthogonal partial least squares discriminant analysis (OPLS-DA) model. The author combined spectral analysis with DEG data to identify specific gene sets, providing an important foundation for the diagnosis and treatment of DLBCL and CLL. This combination helps to further the understanding of the molecular mechanisms of these two diseases and may provide support for the development of personalized treatment plans.

Experimental methods such as RT-PCR and ELISA allow assessment of the effects of genes and proteins such as PIK3CG and IL15 on cell proliferation, apoptosis, and migration. PIK3CG was found to promote the proliferation of B cells. Studies have shown that the proliferative effect of PIK3CG on B cells may be mediated by activation of the downstream AKT signaling pathway. Although its activation usually promotes cell survival, in some cases, excessive activation of PIK3CG may lead to increased cell apoptosis. This phenomenon may be related to the imbalance of intracellular signals, especially under the influence of microenvironment, which may lead to the enhancement of apoptotic signals. PIK3CG may interact with other PI3K subtypes (such as PIK3CD), which may affect cell proliferation and apoptosis. For example, excessive activation of PIK3CD in CLL cells may further enhance cell survival signaling through interaction with PIK3CG. Interleukin-15 (IL-15) is a pleiotropic cytokine that plays a critical role in the immune system. It has complex effects on the proliferation and apoptosis of DLBCL and CLL cells, and these effects are achieved through multiple signaling pathways; for instance, IL-15 binds to receptor complexes on the cell membrane, recruiting and activating Janus kinase (JAK) family members such as JAK1 and JAK3. Activated JAK kinase phosphorylates signal transducer and activator of transcription (STAT), leading to its dimerization and translocation to the nucleus, where it regulates the transcription of related genes. In DLBCL and CLL cells, this pathway can regulate the expression of genes related to cell proliferation and apoptosis, such as the cell cycle proteins and Bcl-2 family proteins mentioned above. IL-15 stimulation can also activate PI3K, which converts phosphatidylinositol-4,5-diphosphate (PIP2) to phosphatidylinositol-3,4,5-triphosphate (PIP3), thereby recruiting and activating protein kinase B (Akt). Akt regulates cell metabolism, proliferation, and survival by phosphorylating various downstream substrates, such as glycogen synthase kinase-3 β (GSK-3β) and forkhead box protein O (FoxO). In tumor cells, activation of this pathway typically promotes cell proliferation and inhibits apoptosis. The mitogen-activated protein kinase (MAPK) signaling pathway is also one of the important pathways through which IL-15 exerts its effects. The signal activated by IL-15 can activate Raf kinase through Ras protein, which in turn activates MEK and ERK. Activated ERK can enter the nucleus where it regulates the activity of transcription factors and influences the expression of genes related to cell proliferation, differentiation, and apoptosis. In DLBCL and CLL cells, the activation status of the MAPK signaling pathway may affect the cell’s response to IL-15, determining whether it proliferates or undergoes apoptosis. These studies all indicate that both PIK3CG and IL15 have complex effects on the proliferation and apoptosis of tumor cells, which are achieved through multiple signaling pathways (73–76).

Relative to the CLL and DLBCL groups, Raman spectroscopy analyses performed herein revealed signficiantly higher peak intensities for the peak positions corresponding to proteins (621, 643, 848, 853, 869, 935, 1003, 1031, 1221, 1230, 1260, 1344, 1443, 1446, 1548, 1579, 1603, 1647 cm^-1^), β-carotene (957, 1155, 1162 cm^-1^), carbohydrates (842 cm^-1^), collagen (1345 cm^-1^), and lipids (957, 1078, 1119, 1285, 1299, 1437, 1443, 1446 cm^-1^) in the control group. Thus, leukaemia patients may be more prone to hypoalbuminemia, vitamin imbalances, dysregulated glycolipid metabolism, and immunological deficiencies. Serological results for these patients noted significantly lower TG, HDL, GGT, ALP, and ALT levels in samples from CLL patients relative to DLBCL patients (P<0.05), suggesting that the metabolic disorders facing DLBCL patients may be more severe such that they face a worse prognosis. The results of this study have the potential to inform the development of Raman spectroscopy-based analyses for the noninvasive screening and detection of biomarkers related to CLL and DLBCL, emphasizing the potential relevance of large volumes of biochemical data to the typing and prognostic assessment of CLL and DLBCL patients. However, the sample size was relatively small and the results may be imperfect. Given the relative rarity of CLL and DLBCL, future studies should undertake more detailed prospective analyses examining the feasibility of classifying CLL and DLBCL more effectively and establishing relevant biomarkers to aid in this process. The present study did not perform in-depth analyses of patient medical history, treatment history, or history of smoking/alcohol intake, which may have impacted the study results. Further large-scale investigations using standardized methods for data collection will thus be vital to improve the accuracy of the analyses. Further age-based stratified analyses are warranted, with an increase in the overall sample size to control for confounding factors.

The potential application of Raman spectroscopy combined with multi-omics techniques in the diagnosis of hematological malignancies: (1) High sensitivity and specificity: Raman spectroscopy can provide molecular-level fingerprint information, distinguishing molecules with different chemical compositions and structures by detecting the vibration modes of chemical bonds in biological samples. In the diagnosis of hematological malignancies, it can detect subtle differences in molecular composition and structure between cancer cells and normal cells, such as changes in biological macromolecules such as nucleic acids, proteins, and lipids, providing a basis for early diagnosis. (2) Non-destructive testing: Raman spectroscopy does not require complex preprocessing of the sample and does not alter the integrity of the sample. It can measure biological samples (such as blood and bone marrow) directly, reducing the likelihood of errors and interference during sample processing, and also facilitating real-time and dynamic monitoring of patients. (3) Comprehensive metabolite analysis: Metabolomics can comprehensively and systematically analyze small-molecule metabolites in organisms, revealing the changes in metabolites during the onset and progression of diseases. In hematological malignancies, there are significant differences in the metabolic pathways of cancer cells compared to normal cells. Metabolomics can identify these specific metabolic markers, providing important clues for the diagnosis and classification of diseases. (4) In-depth understanding of disease mechanisms: By analyzing changes in metabolites, one can gain a deeper understanding of the pathogenesis of hematological malignancies. For example, abnormal activation or inhibition of certain metabolic pathways may be closely related to the onset and progression of tumors, which can assist in the discovery of new therapeutic targets and directions for drug development. (5) Complementary information: The information of molecular structure provided by Raman spectroscopy complements the metabolite composition information provided by metabolomics. Raman spectroscopy can rapidly identify samples with potential diagnostic value, while metabolomics can further analyze these samples in depth, to determine specific metabolic markers and changes in metabolic pathways, and improve the accuracy and reliability of diagnosis. (6) Multi-dimensional diagnostic model: The integration of Raman spectroscopy data with metabolomics data can construct a multidimensional diagnostic model. This model can consider multiple factors such as molecular structure and metabolic changes, and thus comprehensively reflect the characteristics of hematological malignancies, which helps to improve the sensitivity and specificity of diagnosis and achieve early and accurate diagnosis.

Raman spectroscopy combined with multi-omics techniques has broad application prospects in the diagnosis of hematological malignancies, and the identification of IL15 and PIK3CG as potential therapeutic targets also provides new ideas and directions for the treatment of these diseases. Future research should explore the potential applications of these technologies and targets, and contribute to improving the diagnosis and treatment of hematological malignancies. (1) Key role in hematological malignancies: IL15 plays an important regulatory role in the immune system and often exhibits abnormal expression in hematological malignancies such as CLL and DLBCL. Overexpression of IL15 may promote tumor development through various pathways, stimulating cancer cell proliferation, inhibiting cell apoptosis, and affecting the immune microenvironment. Therefore, interventions targeting IL15 would be expected to block tumor growth and spread. (2) Targeted therapy strategy: Monoclonal antibodies, small-molecule inhibitors, and other drugs targeting IL15 or its receptors can be developed to block the binding of IL15 to its receptors or inhibit its downstream signaling pathways, thereby interfering with the proliferation and survival of tumor cells. In addition, gene-editing techniques such as CRISPR/Cas9 can be used to knock out or downregulate the expression of the IL15 gene, achieving the goal of treating tumors. (3) Challenges and prospects: Although IL15 has potential as a therapeutic target, it still faces some challenges in practical applications. For example, IL15 has a wide range of physiological functions in the immune system, and targeting IL15 may adversely affect normal immune function, leading to immune-related adverse reactions. Therefore, future research needs to further optimize the design of targeted drugs and improve their specificity and safety for the effective treatment of hematological malignancies. (4) The importance of the PI3K signaling pathway: The protein encoded by PIK3CG is one of the catalytic subunits of PI3K, and the PI3K signaling pathway plays a crucial role in various biological processes such as cell growth, proliferation, survival, metabolism, and migration. In hematological malignancies, the PI3K signaling pathway is often abnormally activated, and upregulation of PIK3CG may be one of the important reasons for this abnormal activation. Therefore, targeting PIK3CG would be expected to prevent the excessive activation of the PI3K pathway, thereby suppressing the growth and spread of tumor cells. (5) Foundation for research and drug development: Currently, some inhibitors targeting the PI3K signaling pathway have shown positive results in clinical trials, providing a reference for the study of PIK3CG as a therapeutic target. In the future, specific inhibitors targeting PIK3CG could be further developed to improve the efficacy and safety of these drugs. At the same time, the combination with other treatment methods, such as chemotherapy, radiotherapy, and immunotherapy, may achieve better therapeutic effects. (6) Potential problems and solutions: The PI3K signaling pathway also plays an important physiological function in normal cells, so targeting PIK3CG may have toxic effects on normal cells. Future research requires a deeper understanding of the differential expression and functional characteristics of PIK3CG in tumor and normal cells, and the development of more selective targeted drugs to reduce damage to normal cells. In addition, further exploration is needed to investigate the interaction between PIK3CG and other signaling pathways, as well as the resistance mechanism of tumor cells to PIK3CG targeted therapy, to provide a basis for optimizing treatment plans.

No significant differences were observed between groups for certain biochemical indicators. Even with this limitation, Raman spectroscopy could unveil significant differences when analyzing patient serum samples. This suggests that this approach offers greater sensitivity to more conventional peripheral blood analyses, providing an effective screening tool. Another limitation of this study is that patient medical history, medications, smoking status, and drinking status were not considered and may have impacted the results of these analyses. It is thus essential to improve the overall sample size and standardize the data collection approach to achieve greater sampling accuracy. Even so, the metabolomics and statistical analyses employed herein effectively compare differences among samples in a research context and may even have implications for clinical practice, providing a valuable foundation for future research.

## Conclusions

In this study, Raman spectroscopy and bioinformatics approaches were used to differentiate between DLBCL and CLL based on the differential expression of key genes, and the results of these analyses have important implications for the diagnosis and treatment of these two malignancies. Specifically, the DEGs identified herein can potentially serve as diagnostic biomarkers for DLBCL and CLL, and they may further enable the differentiation among DLBCL subtypes to support more effective patient clinical classification. They may additionally offer unique insights into the molecular processes underlying the development of these two types of cancers, as genes that are overexpressed in DLBCL but not CLL may function primarily in the development of the former NHL subtype. The results of these analyses can also help guide the development of personalized targeted treatments, as small molecule inhibitors or antibodies targeting proteins vital to DLBCL cell survival and proliferation may aid in the treatment of this form of cancer. These DEGS can also be leveraged to guide the prognostic assessment of patients with these cancers, aiding clinicians in the selection of the most appropriate treatment plan. Lastly, the bioinformatics analyses performed herein will help unveil the mechanisms through which drugs function in these cancers, potentially facilitating the selection of combination drug regimens. Through comparisons of gene expression patterns between these diseases, genes capable of impacting drug efficacy may be identified, allowing for the optimization of the use of drugs that already exist or the selection of novel drug combinations.

In summary, the bioinformatics analyses of genes differentially expressed between DLBCL and CLL conducted herein can both improve the accuracy of efforts to diagnose these two diseases while also supporting new disease treatment strategies that will contribute to better patient quality of life and treatment outcomes.

## Data Availability

The original contributions presented in the study are included in the article/**Supplementary Material**, further inquiries can be directed to the corresponding author/s.
