# Supplementary material for: Raman spectroscopy and bioinformatics-based identification of key genes and pathways capable of distinguishing between diffuse large B cell lymphoma and chronic lymphocytic leukemia
Source: Front Immunol. 2025 Feb 25;16:1516946. doi: 10.3389/fimmu.2025.1516946 (PMC11893875; doi:10.3389/fimmu.2025.1516946)
Supplement: Supplementary file 1 [file DataSheet1.pdf]

Supplementary Material

Raman Spectroscopy and Bioinformatics-based Identification of Key Genes and Pathways Capable of Distinguishing Between Diffuse Large B Cell Lymphoma and Chronic Lymphocytic Leukemia

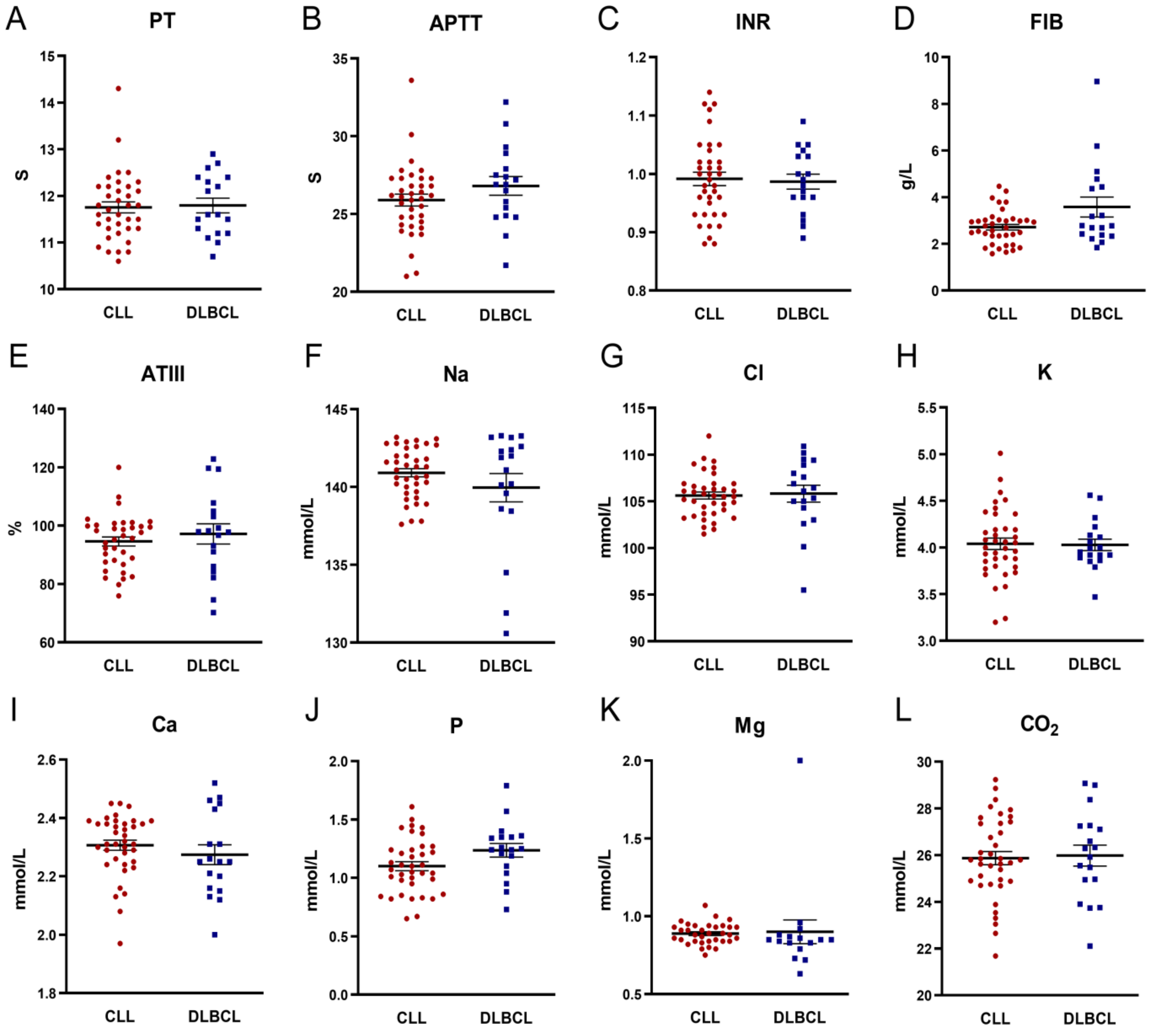

Figure S1 Comparison of peripheral blood coagulation function, trace elements, and other biochemical data between the CLL and DLBCL groups.

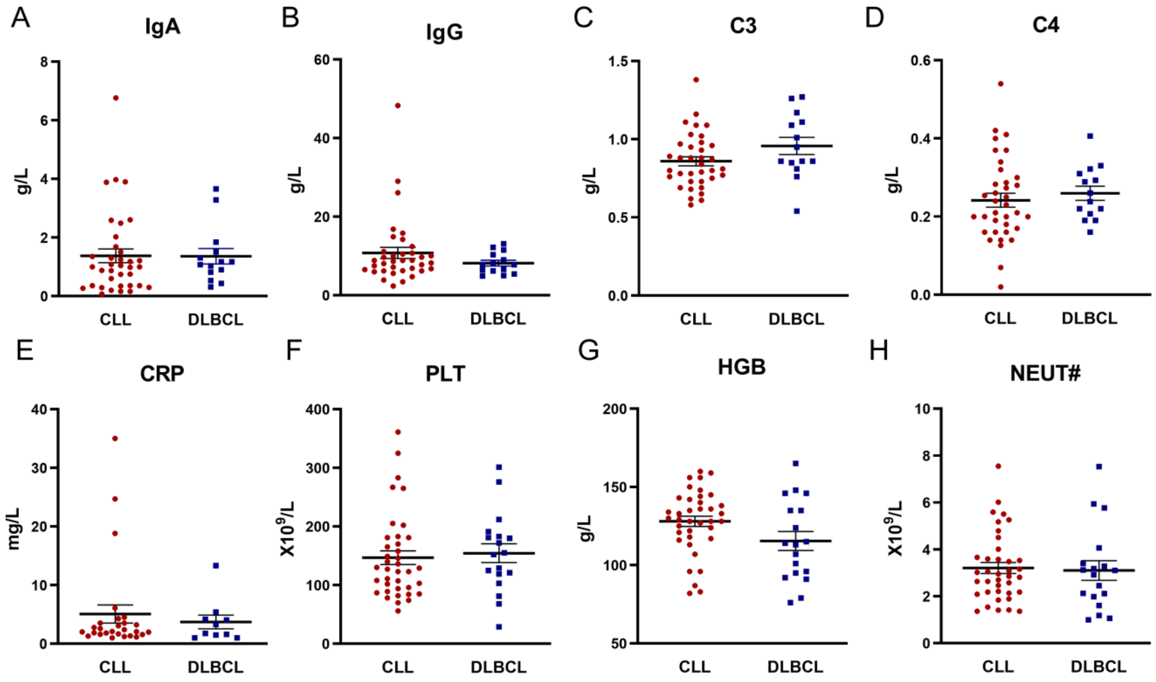

Figure S2 Comparison of peripheral blood immune and hemocyte data between the CLL and DLBCL groups.

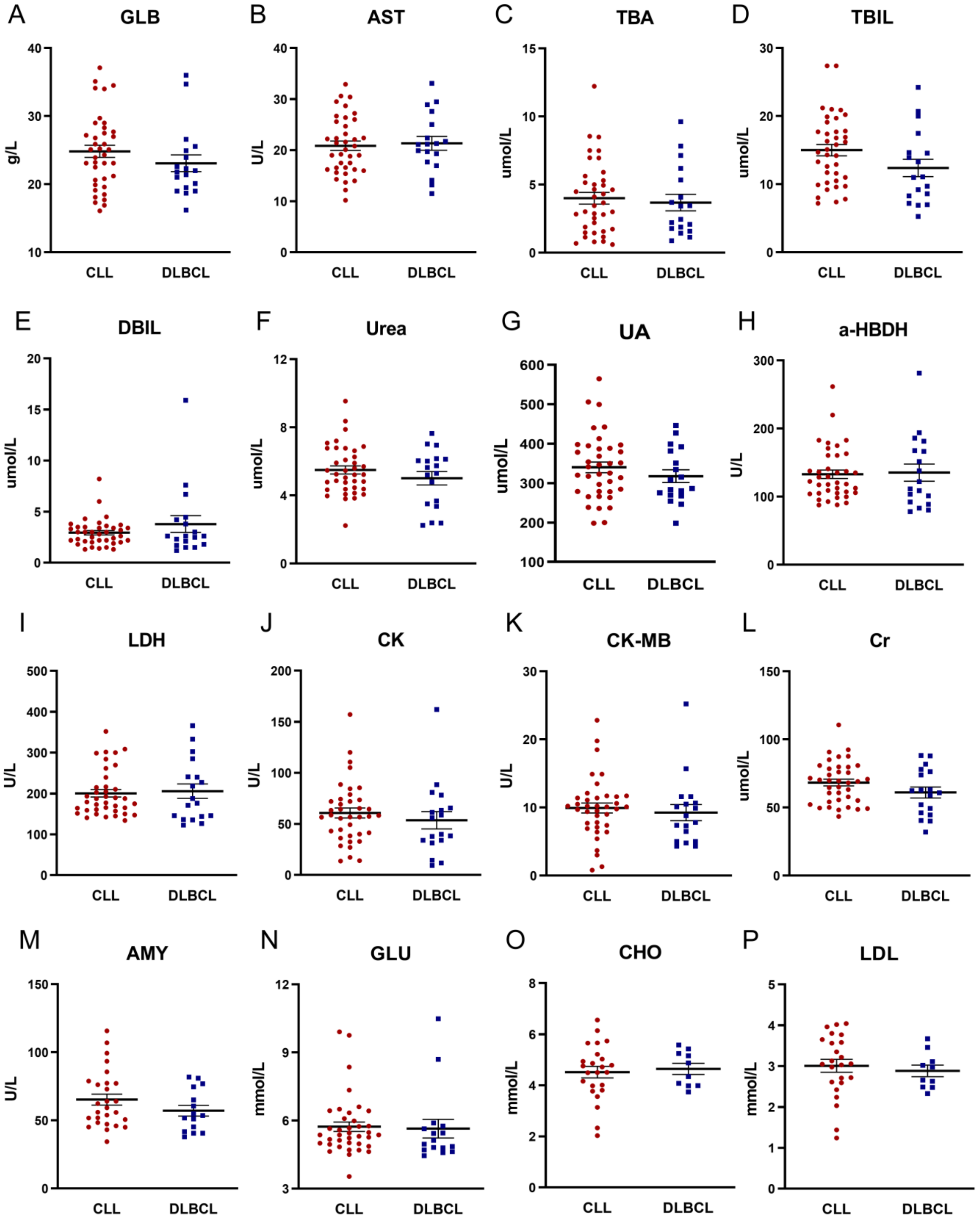

Figure S3 Comparison of clinical and biochemical data between the CLL and DLBCL groups.

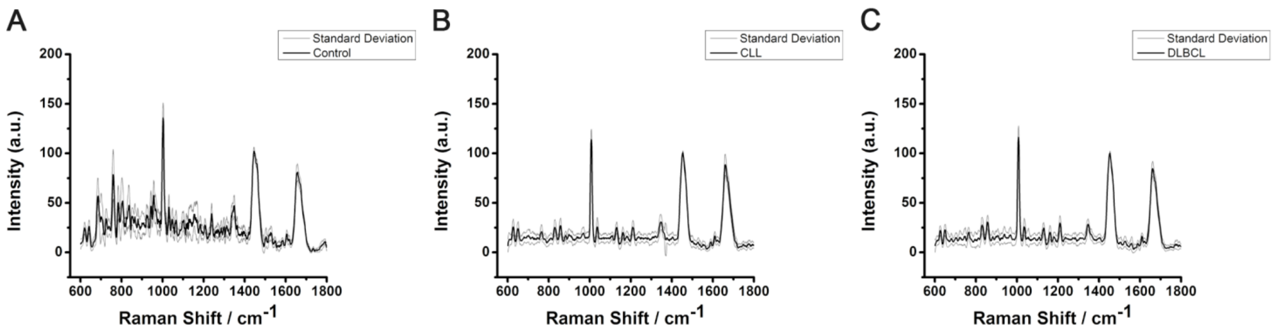

Figure S4 Raman spectra of serum for the (A) Control, (B) CLL and (C) DLBCL groups.

Supplementary Table 1 Comparison of clinical data between CLL and DLBCL groups.

	CLL	DLBCL	*P* value
Sample number	38	18	
Gender (male/female)	25/13	10/8	0.460
Age	59.79±1.548	49.44±3.664	0.0159
WBC	17.195(5.555-49.988)	4.745(3.478-6.073)	0.0002
ALT	14.150(11.600-22.525)	19.460(16.275-35.925)	0.0348
TG	1.070(0.835-1.610)	1.705(1.375-2.105)	0.0072
HDL	1.01±0.057	1.25±0.110	0.0394
GGT	19.250(13.950-26.225)	31.400(19.100-41.653)	0.0079
ALP	76.81±3.416	92.41±6.733	0.0252
TT	17.56±0.190	16.66±0.302	0.0119
NEUT %	18.850(8.050-51.600)	66.700(51.050-76.475)	<0.0001
ALB	68.32±1.120	63.52±2.075	0.0305
a-HBDH	121.500(105.200-160.600)	116.840(91.448-171.125)	0.7577
CRP	2.090(1.590-3.530)	2.525(1.360-4.460)	0.8532
C3	0.86±0.029	0.96±0.055	0.0961
C4	0.24±0.018	0.26±0.018	0.5634
APTT	25.90±0.379	26.81±0.597	0.1914
CHO	4.52±0.224	4.64±0.218	0.7357
LDL	3.01±0.156	2.88±0.140	0.6320
AMY	65.30±4.047	57.16±3.984	0.1969
CO_2	25.87±0.285	25.98±0.448	0.8353
Ca	2.31±0.017	2.27±0.034	0.3419
INR	0.99±0.011	0.99±0.013	0.8022
Cr	68.34±2.474	61.05±4.047	0.1144
CK	60.70±4.849	53.64±8.452	0.4422
CK-MB	10.100(7.325-11.625)	8.300(5.000-10.903)	0.3224
K	4.04±0.060	4.03±0.062	0.9008
ATIII	94.58±1.533	97.17±3.470	0.5025
P	1.10±0.038	1.24±0.058	0.0507
Cl	105.62±0.378	105.82±0.906	0.8374
Mg	0.885(0.840-0.933)	0.850(0.800-0.878)	0.0656
IgA	1.000(0.360-1.635)	1.120(0.748-1.593)	0.4970
IgG	8.710(6.308-11.000)	7.785(5.978-10.600)	0.4321
Na	140.92±0.263	139.96±0.918	0.3277
Urea	5.49±0.235	5.01±0.399	0.2759
UA	340.12±13.491	317.60±16.131	0.3225
PT	11.75±0.119	11.79±0.156	0.8427
GLU	5.370(4.973-6.153)	5.045(4.733-5.730)	0.2487
GLB	24.700(20.425-27.850)	21.900(19.375-25.090)	0.1686
LDH	183.050(158.525-222.000)	181.950(141.825-251.750)	0.8041
NEUT#	2.900(2.168-3.748)	3.005(1.888-3.558)	0.6544
AST	20.86±0.915	21.34±1.367	0.7702
FIB	2.690(2.150-3.025)	2.775(2.368-4.525)	0.1363
HGB	128.03±3.258	115.44±6.087	0.0512
PLT	128.000(92.750-181.000)	153.500(115.000-185.000)	0.4308
DBIL	2.900(2.075-3.500)	2.600(1.775-4.265)	0.9480

TBIL	15.01±0.834	12.39±1.272	0.0857
TBA	4.00±0.432	3.68±0.602	0.6695

Note: Data that conform to normal distribution are represented by M±SE, while data that do not conform to normal distribution are represented by M(Q1-Q3).

Supplementary Table 2 Comparison of spectral data between CLL, DLBCL, and control groups.

	Control	CLL	DLBCL	*P* value
Sample number	5	4	5	
726 cm^{-1}	33.38±1.805	13.46±1.346	14.63±1.321	<0.0001
781 cm^{-1}	46.46±3.730	13.70±0.829	11.82±2.090	<0.0001
786 cm^{-1}	44.38±1.526	11.84±1.276	10.78±1.464	<0.0001
1190 cm^{-1}	21.38±1.182	12.67±0.908	12.01±1.735	0.0007
1415 cm^{-1}	18.05±0.996	12.45±1.442	11.05±1.130	0.0028
1573 cm^{-1}	7.62±0.351	3.87±0.173	4.55±0.965	0.0038
1579 cm^{-1}	11.35±0.676	5.17±0.535	4.72±0.773	<0.0001
1078 cm^{-1}	18.43±1.514	14.78±0.598	13.44±1.232	0.0368
1119 cm^{-1}	27.31±2.637	13.08±1.420	13.97±1.383	0.0005
1285 cm^{-1}	24.02±1.437	14.05±1.616	13.96±1.493	0.0007
1299 cm^{-1}	24.80±2.436	13.45±2.034	12.75±1.180	0.0015
1437 cm^{-1}	86.71±1.124	57.04±1.075	59.95±1.328	<0.0001
1443 cm^{-1}	101.16±0.989	82.29±0.626	84.33±0.947	<0.0001
1446 cm^{-1}	100.12±0.687	91.89±0.515	93.40±0.506	<0.0001
957 cm^{-1}	55.79±3.583	15.79±0.969	14.77±1.256	<0.0001
1155 cm^{-1}	37.57±2.762	15.28±0.380	16.87±2.105	<0.0001
1162 cm^{-1}	32.57±4.342	18.66±0.809	19.14±2.380	0.0130
1345 cm^{-1}	46.96±2.490	30.78±1.302	28.56±1.143	<0.0001
621 cm^{-1}	24.16±1.530	19.06±0.772	15.90±0.976	0.0012
643 cm^{-1}	23.11±1.575	18.02±0.994	16.87±1.395	0.0178
848 cm^{-1}	34.72±1.805	15.24±1.344	17.70±0.896	<0.0001
853 cm^{-1}	36.31±1.442	24.71±1.314	28.85±0.738	0.0001
869 cm^{-1}	28.71±2.658	11.68±0.644	12.44±1.303	<0.0001
935 cm^{-1}	24.89±2.638	14.57±0.811	17.04±1.850	0.0114
1003 cm^{-1}	127.71±2.405	99.87±3.419	102.31±2.064	<0.0001
1031 cm^{-1}	43.37±3.468	19.45±0.617	20.71±1.848	<0.0001
1221 cm^{-1}	15.46±0.750	12.27±1.102	11.36±0.966	0.0202
1230 cm^{-1}	21.44±0.824	14.52±1.596	13.79±0.781	0.0004
1260 cm^{-1}	21.99±1.353	13.44±0.716	12.79±0.957	0.0001
1344 cm^{-1}	45.58±2.451	30.82±1.579	28.39±0.983	<0.0001
1443 cm^{-1}	101.16±0.989	82.29±0.626	84.33±0.947	<0.0001
1446 cm^{-1}	100.12±0.687	91.89±0.515	93.40±0.506	<0.0001
1548 cm^{-1}	11.26±0.945	7.68±0.857	7.06±0.456	0.0048
1579 cm^{-1}	11.35±0.676	5.17±0.535	4.72±0.773	<0.0001
1603 cm^{-1}	18.58±0.847	13.07±0.465	12.83±1.291	0.0022
1647 cm^{-1}	64.03±2.303	49.72±2.276	46.81±1.983	0.0003
842 cm^{-1}	27.25±1.738	12.74±1.460	12.72±0.949	<0.0001

Note: Data that conform to normal distribution are represented by mean ± standard deviation, while data that do not conform to normal distribution are represented by M (Q1-Q3).

Supplementary Table 3 Peak assignments for Raman spectra of serum.

Biochemical group	Peak position (cm^{-1})	Assignments	p-value (respective to peak position)	Reference
Proteins and amino acids	621	Phe		(1, 2)
	643	Tyr		(1-3)
	848	Val, Tyr		(3)
	853	Pro		(1, 2)
	869	Pro		(1)
	935	Pro, Val		(1)
	1003	Phe		(1, 2, 4, 5)
	1031	Phe		(1, 2)
	1221	Amide III		(1)
	1230	Amide III		(2)
	1260	Amide III		(1, 2, 6)
		Collagen		
	1344	CH_2		(1)
	1443	deformation		(1, 4)
		CH_2 bending		
	1446	mode		(1, 2, 4, 5)
	1548	Trp		(1)
	1579	Heme protien		(1)
	1603	Phe, Tyr		(1, 2, 5, 6)
	1647			
		Amide I		(1)
Lipids	957	Cholesterol		(1)
	1078	Lipids		(1)
	1119	Breast lipids		(1)
	1285	Phospholipids		(3)
	1299	Lipids		(1, 2)
	1437	Lipids		(1, 4)
	1443	Lipids		(1, 3, 4)
	1446	Lipids		(2, 4, 5)
Carbohydrates	842	Glucose		(1)
Nucleic acids	726	Adenine		(1, 5)
	781	Nucleotide		(1, 4)
	786	DNA		(1, 5)
	1078	C–C or PO_2		(1)
	1190	stretch		(1)
		Cytosine,		
	1415	guanine,		(4)
	1573	adenine		(1, 5)
	1579	Adenine,		(1, 5)
Carotenoids	957	guanine		(1)
	1155	Guanine,		(2, 4, 6)
	1162	adenine		(2)
Collagen	1345	Pyrimidine ring		(1)

	Carotenoid
	Carotenoid
	β-carotene
	CH_3CH_2 wagging mode

Note: Abbreviations: Phe: phenylalanine; Tyr: tyrosine; Met: methionine; Pro: proline; Hpro: hydroxyproline; Trp: tryptophan; Val: valine.

Supplementary Table 4 Common DEG identified in GSE57083 and GSE68950.

Gene symbol (up-regulated DEGs)
ZNF117, ZNF665, ASAP1-IT1, LPP, ANP32A-IT1, NAIP, SCAF4, PRKD3, OSBPL10, KANK1, RAPGEF5, TRANK1, METTL7A, LTBP1, LOC100505915, PIK3CG, ANKRD36B, MXI1, FADS3, SLA, CUX1, S100A10, ZHX2, RALGPS2, PRKACB, KMO, SYK, CD180, DNAJC12, WASF1, CD72, ANKMY2, ARHGAP25, BCL6, BZW2, STK39, GPR18, BACH2, CD24

Supplementary Table 5 Common DEG identified in GSE57083 and GSE68950.

Gene symbol (down-regulated DEGs)
VIM, PLA1A, CCND2, HERC5, EBI3, DSE, IER3, TIMP1, BCHE, ARNTL2, SPRY2, CCL4, YBX3, CRY1, GSDME, TNIK, APOBEC3B, TUBB6, RGS1, ITM2A, ADGRE1, DRAM1, STEAP1, CCL22, FERMT2, CKAP4, GALNT11, THEMIS2, ANO10, FCER2, ZBTB32, IL7, TSPAN12, PON2, IL15, FSCN1, CORO1C, LHX2, IL12B, ANO3, CD58, ASS1, TNFRSF8, VASH2, FHOD3, OXTR, TNFRSF14, LGALS1, INAVA, GADD45B, CYP1B1, CCL5, ACTN1, CD300A, PARVB, BHLHE40, PIR, MCTP1, NACC2, P2RY10, AQP9, CYBRD1, IL6ST, IFI35, ACKR3, MUC13, PTP4A3, RDX, BLVRA, GEM, EGOT, INPP4B, MAP1B, CCL20, VCAM1, IRAK3, MSC, PTPN13, APOL3, SPART, SEPTIN8, HSPA4L, APOL1, HEY1, IFITM3, DACT1, FRMD4A, IFITM2, BMP2, TRIP10, SPARC, APOBEC3G, APP, GNA15, TNFSF14, TMOD1, ITM2C, PRKCH, IL1R1, DDB2, UST, SIX3, IGFBP4, PRR5, RXRA, PBX3, MEOX1, GNAQ, BOLA1, FBN1, CTNNA1, NMT2, TUSC3, KCNK5, RAB9A, IL27RA, FTH1, ARHGAP6, ME1, APOBEC3C, TPM4, FDXR, FLNA, LMNA, TNFSF9, UNC119, RNF19A, PLD1, PPP1R26, PDE8A, EFNA5, COPG1, DLG5, SEC61A1, P2RX4, GRAP2, ADAM8, CDC20, COLEC12, RIN3

Supplementary Table 6 DEGs of DLBCL vs CLL identified in GSE57083.

Gene symbol (up-regulated DEGs)
LOC284898, GMCL1, RPS11, CCDC28A, ZNF117, ZNF260, CCDC146, ZNF862, ZNF665, LOC286437, MIA2, PNPLA8, HIP1R, NUMB, LINC01410, PAN3, TSACC, AGAP4, MIRLET7D, ADARB2-AS1, PHF20L1, KHDC4, SLC38A9, MAST4, PABPC1L, POLQ, ZFP69, SERAC1, NOTCH2, KLRB1, ATP6V1FNB, TRIM52, TLR9, RFPL3S, CYB561A3, BTBD6, SEMA5B, SETD7, ASAP1-IT1, VPS4B, LOC100190986, ERP27, SEC31B, LPP, WDR66, DOK7, EFHC1, MGARP, C11orf80, ZNF561-AS1, ZNF585B, NPTN-IT1, RBM4, ARHGAP44, CIDEB, LINC01806, ZNF585A, GUSBP4, FGD4, ADAM1A, FAM214A, PIP5K1B, OFD1, LINC00342, NSUN6, LEAP2, DMTF1, FAM229B, TRIM13, SBF2, KHDC1, REV3L, LSM8, TGFBR2, HRK, RSKR, ZNF431, ZSCAN16, MYD88, YPEL3, COX10-AS1, ZCCHC7, ADAM28, WDPCP, TRMT13, LRRC10B, OCIAD2, METTL2B, NAIP, SLC26A6, ZBTB20, SESN1, ANP32A-IT1, HINT3, ZFAND6, DLEU2, SNX29, FOXO1, CILP, AKAP9, DENND1B, LINC00954, PSMA3-AS1, ABHD18, SCAF4, BTBD18, PRKD3, OSBPL10, KANK1, RAPGEF5, EPM2AIP1, PODXL, TRANK1, MED13L, LEF1, ST14, RABGAP1L, ZNF512, PSMD6-AS2, METTL7A, MCTP2, CCZ1B, LTBP1, LOC100133315, FOXO3, TAPT1-AS1, LOC100505915, SIGLEC10, ANKHD1, HOXA5, SRGAP2, KANSL1, HOXA10, IFNLR1, SMG1P5, PMS2P5, ARL15, UHRF2, LINC00597, MAP3K20, BEND4, RHOH, CCDC91, FADS3, RBMS1, MIR3916, LOC114224, SLA, NOTCH2NLA, RNFT1, ZNF608, CUX1, ZEB2, PPP3CA, ANKRD36B, SERPINB1, KCNMB3, RCAN3, S100A10, PIK3CG, ZHX2, RALGPS2, BCL2L11, PRKACB, FTX, PLAG1, KDM7A, KMO, LRMP, SYK, ANKRD36BP2, MXI1, CD180, DNAJC12, EML6, RNF144B, WASF1, DTX1, RUNX1-IT1, CD72, UGT8, PLEKHG1, JAZF1, LOC100505501, ANKMY2, ARHGAP25, FOXP1, GPR160, BCL6, BZW2, STK39, GPR18, TSPAN13, BACH2, CD24

Supplementary Table 7 DEGs of DLBCL vs CLL identified in GSE57083.

Gene symbol (down-regulated DEGs)
BCHE, PLS3, SPRY2, CRY1, IFI44L, TUBB6, ITM2A, FERMT2, CCND2, ROBO1, GALNT11, UNC13C, TCEAL4, HERC5, FSCN1, BNIP3, IFITM1, EBI3, CHMP4C, DMKN, LHX2, STEAP1, GSDME, CCL4, FHOD3, ASS1, APOBEC3B, INAVA, PLA1A, IL7, PFN2, ANO3, NEXN, GAS2L3, IFI44, SERPINH1, CMPK2, CYP1B1, TIMP1, RAB31, PRTFDC1, LGALS1, CBLN2, IFIT3, CCL22, IL12B, OXTR, PIR, PTGFRN, PKHD1L1, PDGFRL, TSPAN12, FJX1, DRAM1, IFI35, CPNE8, FCER2, GAS2, FLRT3, NACC2, BTLA, SCML1, CXXC4, CCL5, TNIK, BHLHE22, GEM, NDN, IGHD, IL15, PRRT3, RTL6, KL, LY6E, MAP1B, VCAM1, PHGDH, TCEAL3, SELENOM, MSC, CD300A, CD226, LINC02381, LYSMD2, IL4I1, ANO10, INPP5A, ALKBH3, MSX1, HSPA4L, DNM3OS, THEMIS, SLC16A9, SLC2A3, MB21D2, MCTP1, IER3, IFITM3, SERPINB10, BEX2, PRXL2C, CCR10, IFITM2, FAM171A1, THEMIS2, KIF21A, SPARC, ZBTB32, SLCO5A1, VLDLR, FAM171B, CTSV, ARMCX2, DACT1, PLOD2, CD274, IL18R1, HEY1, LINC01588, B4GALT6, ALX1, PRKCH, CRTAM, BHLHE40, DDR2, UST, LOC101928429, NME4, CBS, STAT1, EFNB2, APP, RILPL2, BMP2, EGOT, NFIC, IGFBP4, CYBRD1, LOC101926963, BSG, ALPK2, YBX3, P2RY10, PCBD1, LINC01619, ADGRE1, ANO5, RNF157, RGS1, BIN1, APOL3, TNFRSF1A, KCNN2, LYPD6B, PRR5, LINC02363, CD163L1, PARVB, EOMES, RXRA, PON2, BLVRA, GPR63, CKAP4, POGLUT3, TEAD4, DCBLD1, LAMC1, MEOX1, ARNTL2, CDH10, TNFRSF8, POGLUT2, DLX5, CCL20, GNAQ, BOLA1, CD58, ITM2C, ACTN1, MSC-AS1, RPL26L1, NEXMIF, PIMREG, GLT8D2, VIM, FBN1, STX11, PRRX1, LGALS2, PIK3R6, ACTN3, MEIS2, PYGL, DNAJC14, GADD45B, BST2, C10orf99, TRIM6, STC2, TWIST1, LHX8, NKIRAS1, PBX3, CENPB, TMEM51, ATP8B2, F12, IL1R1, GPR84, KCNK5, SIX3, LRRTM4, OASL, IRAK3, CFH, ARHGAP6, TNFSF14, ME1, SHISA8, APOBEC3C, SEPTIN8, MUC19, SLC10A2, PRDM13, PXDNL, C8orf88, SLC25A23, VASH2, NMT2, LNX1, PTX3, RAB38, NUPR1, MUC13, TNNC1, ERO1A, C20orf204, STEAP3, CDIP1, NAGA, PILRA, SLC37A2, GNA15, TNFSF9, LRP12, MFAP2, KCND2, NEURL3, CDC42EP5, ASL, UTS2B, PLK1, RHOV, LPGAT1, NR4A2, NRROS, FDXR, FADS2, KANK4, PLD1, TPM4, FRMD4A, NID2, LIN28A, ARHGAP28, EPB41L3, TMEM45A, TRIP10, TUSC3, EIF4G1, CORO1C, RORA, ZG16B, FTH1, NAP1L2, EFNA5, PIGR, M6PR, ZEB1, DPP4, PSTPIP1, CCR6, VASP, DUSP7, ETV4, SH3RF1, TRIM71, RILPL1, CDC42EP1, STIP1, FAM92A, COPG1, PTP4A3, LOC154761, H3C2, PPEF1, CEBPD, MMP3, MAPKAPK3, SUOX, GNGT2, C15orf48, MRPL2, TMEM158, MAB21L2, IL27RA, NABP2, MEIS1, PDE8A, TMEM273, ALYREF, EMILIN1, ACKR3, CAPN8, STT3A, SPRY1, ARL13B, AQP9, KRTAP17-1, TMOD1, RDX, ALDH1L2, MLEC, PPP1R26, LAMA1, SOCS3, LAP3, DCPS, CMTM3, SPIN3, MATN1-AS1, RRAS, GNPNAT1, VMO1, LOC729970, LMNA, SH3RF3, COL3A1, KLHL13, FLNA, AP3D1, APOBEC3G, TCF15, FUT11, RIOX1, CDC42EP4, DLG5, TRIM72, SLIT2, NME3, TMEM220, RAB9A, SLC17A6, CHCHD10, NID1, ZNF35, ELFN1, PARP3, DLX2, MRPL17, C12orf54, ATP6V1A, CPEB1, TNFRSF9, STOM, HSD17B14, PTPN13, PPDPF, ARHGAP10, IL6ST, SOAT1, HNF4G, ALDH1B1, SPART, PDE10A, KDELR2, HES4, PTAFR, ERO1B, H3C7, SNRNP40, TNFRSF10D, MTERF2, FOXG1, EEA1, DNAJC17, USO1, SELENOS, PROS1, BLVRB, ACOT7, TPBG, FSD1L, ODF3B, ARID3B, SP2-AS1, ARHGAP21, USP40, H2AC16, UNC119, HECW2, FBXL8, GDF15, DSE, TNFRSF14, PPIB, SLC50A1, FNDC10, SV2A, MACROH2A2, TET2, DOCK7, NRBP1, WASHC4, FLOT2, COL16A1, LMNB2, DAG1, ADAM8, ALMS1-IT1, TMEM47, PRRT2, IGFALS, TMED2, CXCR3, THNSL1, GMPPA, APOL1, SPACA3, RIPK2, P2RX4, OLFM3, BOLA3, LRRC4B, PRR7, GRAP2, CPPED1, HACD2, DUSP16, SLC6A8, TSPAN4, NINJ1, TLCD1, LSM12, ANXA6, LOC100506563, NBAT1,

WWTR1, DHX58, LYAR, CABP1, CDC20, TMEM151A, MTUS1, FKBP10, ABCA3, INPP4B, YWHAE, BCL2L10, PEX10, C2orf76, PAK1, RAD54B, DENND1A, LINC00467, FAM9C, FOXO4, SRBD1, SEC24C, FBXL7, RNF19A, LINGO1, ORC1, PRPF4, EPHA7, FAM110D, RBPMS, LILRA4, CCDC8, NEFL, ZBED1, PDZD8, SIK1, ACTA1, DUSP3, ADGRD2, AGRN, ADAMTS3, SDSL, ARHGEF25, PRF1, RDH10, COLEC12, SCAF1, DDB2, LARS2, ECD, ARHGDIA, MSI1, STARD4, RNASEH1-AS1, TUBBP5, LOC100506446, PTGER2, CTNNA2, CTNNA1, RGS14, PAK1IP1, RIN3, CTTNBP2NL, COL1A2, SEC61A1, PRICKLE3, TAC1, NFKB2, CHCHD6, C19orf81, SNAPC2, RASSF3

Supplementary Table 8 DEGs of DLBCL vs CLL identified in GSE68950.

Gene symbol (up-regulated DEGs)
CDK2AP1, PSKH1, ZNF432, CFAP298, C20orf27, CPNE1, MAP3K7, HOOK2, ROCK2, MAPK1, HDDC2, RMND5A, ITGAE, RFXANK, JADE1, POLD1, IPO7, STS, TECR, HSD17B12, TTC19, MCM3AP-AS1, VPS8, KDM6B, HMBOX1, PEX1, CYP39A1, UBE2C, FAM216A, RHOT1, TCF4, ZCCHC2, ZNF580, POT1, HDAC7, JUND, RANGRF, TSPYL2, POLR3G, PREPL, MKKS, USP6NL, OAZ2, RNASEH2A, MSH2, CLASP2, ASAP1-IT1, SWAP70, GID8, ACTR3B, PPP6R1, PHKA2, TERF2, ZNF8, GALNS, AFF2, TRIP13, SMARCD2, PKN1, APBB1IP, BRWD1, SCAND1, BCOR, MAP3K3, ZKSCAN4, KRCC1, NBPF1, RNF170, RPGRIP1L, RNF187, KIZ, INTS8, RPRD1A, DYRK2, NOSIP, DVL2, LTA4H, VAMP1, TOP1, DESI2, POU2F1, EZH1, DCUN1D2, EIF4G3, BBOF1, SMARCD3, RPIA, CTNS, CHD8, DEPDC1, UGGT2, CAMSAP2, PSRC1, SDHAF3, TTPAL, USP13, BIRC5, AUH, TMEM231, CDKN3, ALG6, CDC20, AGBL5, ZNF571, MAP1LC3B, ABCA11P, TAF12, SGF29, ZNF117, TOGARAM1, CHMP7, C1GALT1, TRIM8, MADCAM1, MTMR2, ANP32A-IT1, ZNF271P, PICALM, PLA2G12A, PPP2R1B, ASIC1, FAM49B, HS2ST1, SAMHD1, HMGN1, PRKCI, CHEK2, ZNF665, CBFA2T2, IMP4, HSD17B6, DHFR, PAQR3, MLXIP, PGAP1, COCH, ELOA-AS1, PTDSS1, SCRN1, KLHDC2, MPHOSPH6, NUDT1, METAP2, HMCES, SLC25A37, FBXO41, NUBPL, TNKS, NAIP, SSBP2, CEP83, ZDHHC4, SCAF4, TMEM106B, WDR60, TAF4B, ABL1, RGL2, CNTRL, ZNF592, ZNF107, RFK, ENTPD4, ZNF93, SOX12, IFT52, SCAMP1, MIS18A, CTNNBL1, SHOX2, PPP2R5C, RBM4B, CEP68, TRAPPC2, E2F5, ACSF2, UBE2S, ENDOG, KBTBD2, DKFZP586I1420, SIRT1, SOGA1, OGFRL1, ALMS1, MEN1, CDK14, RAP1GDS1, MYO9A, VAT1, TBCC, PHLPP1, CPNE3, NDRG3, PBK, THAP12, PPM1F, C11orf95, GAMT, SSX2IP, TOB2, CNP, FILIP1L, PIGN, ZNF43, MAPK12, LRRC37A2, PDLIM2, CERS2, ZNF430, TMEM135, TUBB3, METTL7A, NET1, STAT5B, MAP3K1, CCNG2, CCND3, WEE1, PRKD3, PCDH9, SPTLC2, DCXR, BCAS4, SEPHS1, PCMTD2, OSBPL8, DBN1, SCCPDH, PHC1, ETS2, RCN2, PFKFB3, LOC100996756, MRGBP, SLC35A1, BLCAP, CD81, PIK3CG, TSPAN3, CCDC69, RFC3, ATM, ANKRD36B, DNAJC10, SIGLEC15, MXI1, ZNF395, CLSTN1, HGSNAT, CHD3, DUSP14, REXO2, VGLL4, ZNF652, FAF1, SNHG17, DET1, C11orf49, SNAPC1, RPL39L, MEF2A, SLC35D1, YPEL1, HDAC1, RASSF2, MCUB, PRCP, DEPTOR, CYFIP2, ZMYM1, TMEM50B, ZNF573, SLC25A15, POLE3, CD37, MTUS2, KIAA0355, SPI1, HLA-DOB, TMEM123, RABAC1, SLC39A4, PAFAH1B3, ALOX5AP, PSPH, QRSL1, NLK, IFT57, FAM53B, SNTA1, ZNF85, PHC2, SULT1A2, ASAP1, RCBTB1, ZKSCAN7, SLCO3A1, PCYOX1, ZNF83, ZNF137P, DHX40, PTPRC, NECAB3, CASD1, GALNT1, RCOR1, MNAT1, PLEKHA5, SLC43A1, RGS19, GPD1L, PLCXD1, RBFOX2, AMFR, MEST, GLIPR1, RAB11FIP1, ESR2, SLC38A6, ASF1A, KCNMB4, GCHFR, TMEM100, ARHGAP5, DCK, LHPP, ABCC5, RSAD2, PEG10, TMEM156, MGST2, RBM38, MAPRE2, APLP2, FGD6, PKIG, TM7SF2, PHF10, ZNF106, BRD3OS, CCDC88A, ZNF91, ALDH5A1, RHOBTB2, ASB13, GDPD5, SLC16A6, CREG1, BPTF, CAND2, FADS3, MARCHF1, OSBPL10, LPP, FCGR2B, TRANK1, SULT1A1, TXNIP, LCK, MEGF9, MARCKSL1, SOX9, PLEKHF2, HECA, SYPL1, OSBPL3, PTPN18, SYBU, TMEM159, LYL1, TOX, MLLT11, GNAZ, KIF5C, LRP4, RALGPS2, KMO, ALOX5, CD180, SYK, RGS2, ZNF532, PITX1, PTK2, LOC100505915, S100A10, TCEA2, CD79B, CD72, WDR19, RAPGEF5, CD247, SERPINF1, CUX1, ALDH2, MFHAS1, MGMT, XYLT1, HTR3A, SLA, SEL1L3, MME, ABLIM1, TCL1A, SLC6A16, MILR1, ZHX2, ITPKB, ARHGAP25, BCL7A, STAP1, NELL2, PRKACB, FCRL2, RGCC, NRN1, KCNK12, BACH2, WASF1, LTBP1, GPR18, MYBL1, OAT, SOBP, BCL11A, ELL3, STK39, ANKMY2, KANK1, DNAJC12, PDGFD, BCL6, TNS3, TRIB2, BZW2, CD24, RGS13, VPREB3

Supplementary Table 9 DEGs of DLBCL vs CLL identified in GSE68950.

Gene symbol (down-regulated DEGs)
EPS8, CCR7, LY75, VIM, IL2RB, LGALS3, PLA1A, SLAMF1, SRGN, CCND2, ATP1B1, NCF2, TNFAIP3, CREB3L2, FGR, HERC5, EBI3, DSE, IER3, TJP2, PLAC8, MREG, TIMP1, SQOR, CYFIP1, CDKN1A, KCNK1, RBM47, PDLIM1, LITAF, BCL2A1, NCALD, LGMN, SAMSN1, MAP3K5, CELF2, BCHE, IPCEF1, TNFRSF17, ARNTL2, MID1IP1, ANXA4, CCL4, RGL1, YBX3, ENTPD1, SLAMF7, GSDME, TNIK, APOBEC3B, MARCKS, CD200, TENT5A, DTNB-AS1, TUBB6, CD69, TNFRSF1B, BATF3, CD48, RGS1, CD80, TRAF1, BATF, ADGRE1, SIDT1, GMFG, IGHM, DRAM1, ADGRE5, RND3, STEAP1, CCL22, CLIC2, TMEM140, S100A6, CD44, CLEC2B, CKAP4, DUSP2, CST3, PTPRK, IL17RB, FAM117A, CTSH, ITM2A, S100A4, CAPN2, THEMIS2, ANO10, FCER2, NCK2, INPP1, HSPB1, IL21R, ZBTB32, IL7, TSPAN12, MYO6, DSG2, MGLL, FCMR, ENPP2, MAGEA6, PRKCB, PON2, TSPO, CPOX, IL15, FNDC3B, MNDA, GPR137B, FAS, CAPG, CORO1C, LHX2, IL12B, HLA-F, ADGRA3, ANO3, IL32, DUSP5, RCN1, CD58, ASS1, GPX1, IL2RG, TNFRSF8, MDFIC, FSCN1, CSGALNACT1, LOC728392, AOC1, AICDA, VASH2, LMO3, SKAP1, ZBTB38, SEMA4D, TES, ATF3, TRIB1, MVP, FERMT2, OXTR, TNFRSF14, OPTN, LGALS1, IER5, EVI2B, ACSL1, CADPS2, ADA, NOD2, CLIP2, GADD45B, SPRY2, CCL5, ACTN1, GLRX, FARP1, CD300A, ARL6IP5, PARVB, FEZ1, AHNAK, IL15RA, ENC1, MLLT3, BHLHE40, ERV3-2, FOCAD, GALNT11, FHOD3, MACF1, IGSF3, SGK1, CASP1, ZMAT3, MYO1E, PIR, MCTP1, OCA2, NACC2, CD9, ABCA12, PIM2, PIEZO2, P2RY10, ARID3A, SELPLG, AQP9, CYBRD1, INAVA, IL6ST, CYB5R2, GBP1, CNN3, BAMBI, FAM169A, ICAM1, DUSP22, ACKR3, MUC13, GPR15, ARNTL, PTP4A3, PRDM1, AHR, RDX, CERS6, BLVRA, MOCOS, KIFAP3, IRF4, DUSP10, DTX4, NMI, EGOT, RAB29, DOCK10, INPP4B, SSX1, DNASE1L3, SYNPO, TAPBPL, C5orf30, NFKBIA, SLC1A1, BIRC3, CCL20, IRAK3, TRAF3IP3, TDO2, NFKBIE, GSE1, PTPN13, DCTD, IFIH1, IGFLR1, PIEZO1, CD86, ARHGAP17, SYNGR2, APOL3, WNT5B, VCAM1, BCAS3, GBA3, ETHE1, SPART, MAP1B, SEPTIN8, ABCA5, STAT3, CRY1, KHDRBS3, STK38L, CD59, APOL1, HEY1, ANXA1, CHST12, DACT1, FRMD4A, BMP2, TRIP10, ARHGEF3, TFPI2, XBP1, GUCY1A1, SLC12A7, LAT2, APOBEC3G, LAX1, APP, UGT2B17, GLDC, GNA15, SGMS1, ALCAM, GATM, TNFSF14, GLUL, PIP4K2A, ECHDC2, SIPA1L1, TMOD1, MICA, FHL2, ETV6, CYTH1, COL9A2, TRAC, ITM2C, IL1R1, DMD, HSPA6, ZDHHC14, GSAP, CBX7, SPOCK1, DDB2, ISCU, STAP2, CD70, BACE2, CD40, SIX3, CTSO, CDC42EP3, HIPK2, SPATS2L, TRPV2, VOPP1, TESC, TOM1L1, PAM, GVINP1, MAGEA12, SUB1, LAMA3, EGR3, IFIT5, TACC1, WDR91, SLFN12, RXRA, HOXC6, HMOX1, EFR3A, HSPA4L, ACTA2, MAN1A1, UPB1, KYNU, PBX3, TIAM1, PMAIP1, CLDND1, BTN3A2, CALHM2, DNAJC1, IFI35, GNAQ, CHST7, NLRP1, S1PR1, FBN1, ITGAV, PRKCH, MAPK6, GALNT6, IRF2, USP3, RASGRP3, TNFRSF13B, CTNNA1, MEOX1, SOCS2, PECR, CD82, B4GALT5, WIPI1, SMAD7, NMT2, CYP1B1, RAB27A, RIMBP2, TUSC3, IGF2R, CHFR, CRYZ, RAB9A, SSR3, IL27RA, IRF1, GBP2, IVL, DNAJB9, LGALS14, FTH1, CACNB4, MSRB1, LCP2, UST, PHLDA2, ITGB7, TPM4, CD53, GADD45A, CFLAR, ARFGAP3, GPHN, TFRC, HNF1B, KCNMB1, FDXR, NFE2L3, IRF5, ARID5A, FLNA, CYTIP, SH2D2A, LMNA, MAL, F2R, HYOU1, SEC24A, UNC119, ARHGEF5, LCP1, IFITM2, ME1, TCF7, INSR, SLC19A2, PLAAT3, CASK, TXN, PASK, RAB8B, RNF19A, HGF, GCA, MSC, SPARC, CRYL1, ZNF267, PPP1R26, RPS27L, CHAC1, TRIO, EMC2, BTN3A3, UGCG, SLC43A3, SNX11, GPSM3, PLP2, SPTBN1, MGST3, PDE8A, B3GNT2, KLF11, EEF1A2, ZMYND11, HLA-DQA1, NABP1, FAM111A, GFPT1, P4HA2, CCDC88C, CDYL, CTC1, RGS3, NFKB1, MSL3, C15orf39, NOTCH1, PTPN7, USP12, BCL2, KCNK5, LYN, ATOX1, ZC3H7A, S1PR4, ELL2, KLF10, SEC31A, SNX4, JADE3, RALB,

FASTKD1, JUNB, NEDD4L, TNFSF13, GEM, MAN2A1, IGFBP4, ETNK1, AFF1, SERP1, APOBEC3C, GSTT1, DLG5, PARD3, ZYX, RAC2, ARL2, AGAP1, SLC12A6, SMCO4, PLXNC1, RRM2, MAGEH1, USP48, GSTM2, ZFP36, MFN1, RHOQ, CTH, CALCOCO2, SEMA4C, RUNX3, PDGFA, TNFSF9, TGM5, KIAA0754, RYBP, TNIP1, IKZF2, RAN, GSTM1, LSP1, MBD5, SIL1, RHOB, NAMPT, CSRP1, PAOX, ANXA7, PLXNB2, PTGER4, LRCH1, KCNN4, ARHGAP6, EMID1, PPP1R15A, TNFAIP8, HSD17B8, RUFY2, SEC61A1, HCAR3, MANEA, P2RX4, STK10, PLEKHF1, RAB40B, LXN, ADAP1, MMP7, MYO1D, ABHD4, CARMIL1, DENND3, CEMIP2, BTN2A2, DBI, LSR, COPB2, YES1, GRAP2, C12orf4, KAZN, CDK5R1, RTP4, GSTM3, CXorf21, SOCS1, ADD1, COMMD3, SPCS3, RASSF4, ARCN1, EFNA5, TICAM1, SEC61B, TYMP, TRIM5, IGF1, TCIRG1, GALNT10, INPP4A, TNFAIP2, CREBL2, MYH9, FBXW7, DEPP1, PLD1, COLEC12, TAP1, RIN3, TNIP3, IFITM3, EHD4, PYHIN1, SERPINB2, BTN3A1, IL3RA, KIF13B, ADAM8, MRPS28, BOLA1, DMXL2, CHMP2A, NOC3L, SLC2A4RG, LPCAT1, ITGB5, PPCDC, COPG1, PRR5, RNF4, NDUFA6

Supplementary Table 10 Top 5 GO Enrichment Analysis Results for the DCBCL vs CLL comparison.

Category	Term	Count	%	P Value	Genes
Biological Process (BP)	immune response	19	10.6741573033707	2.75E-07	IFITM3, IFITM2, CCL22, TNFSF14, IL1R1, IL15, CCL20, GEM, PIK3CG, IL27RA, RGS1, IL7, CCL5, FTH1, CCL4, TNFSF9, ACKR3, TNFRSF14, CD24
	inflammatory response	16	8.988764045	3.12E-06	CCL22, IL1R1, IL15, CCL20, IGFBP4, CD180, STK39, PIK3CG, BMP2, THEMIS2, BCL6, CCL5, CCL4, ADAM8, APOL3, NAIP
	cellular response to tumor necrosis factor	10	5.617977528	5.06E-06	VCAM1, CCL22, CCL20, CCL5, CCL4, CYP1B1, CD58, YBX3, ASS1, GSDME
	cellular response to interferon-gamma	8	4.494382022	2.42E-05	CCL22, CCL20, CCL5, CCL4, IL12B, CD58, VIM, ASS1
	positive regulation of GTPase activity	10	5.617977528	4.00E-05	CCL22, RGS1, CCL20, CCL5, CCL4, RAPGEF5, ARHGAP6, FERMT2, S100A10, RALGPS2
Cellular Component (CC)	cytoskeleton	21	11.79775281	2.77E-08	TMOD1, FHOD3, KANK1, TPM4, RDX, STK39, VASH2, FRMD4A, PARVB, PTPN13, CKAP4, OSBPL10, SEPTIN8, FSCN1, TRIP10, CTNNA1, SPRY2, TNIK, VIM, WASF1, FERMT2
	plasma membrane	75	42.13483146	4.84E-07	IFITM3, KCNK5, APP, OXTR, SPARC, IFITM2, TUSC3, AQP9, TSPAN12, PIK3CG, IL27RA, ADGRE1, RGS1, GRAP2, ME1, TNFRSF8, MUC13, PRKACB, RALGPS2,

				PRKCH, SYK, CD300A, IL1R1, ACTN1, CD180, IRAK3, CKAP4, GEM, ANO3, FCER2, PTP4A3, MAP1B, ADAM8, RAPGEF5, IL6ST, STEAP1, COLEC12, KANK1, PON2, EBI3, EFNA5, PLD1, LPP, GNA15, CTNNA1, FLNA, ANO10, TNFRSF14, CD58, BCHE, P2RY10, CD72, VCAM1, TNFSF14, RDX, CYBRD1, PARVB, PTPN13, SPART, BMP2, P2RX4, DLG5, GNAQ, TRIP10, SPRY2, RAB9A, TNFSF9, ACKR3, NMT2, VIM, ITM2A, GPR18, FERMT2, GSDME, ITM2C
	endoplasmic reticulum lumen	11	6.179775281	2.50E-04	BCHE, APP, LGALS1, IGFBP4, EBI3, IL12B, TIMP1, APOL1, LTBP1, CKAP4, FBN1
	cytoplasm	68	38.20224719	4.12E-04	FHOD3, APP, SPARC, IFI35, BZW2, ARHGAP6, YBX3, MEOX1, PIK3CG, HERC5, TUBB6, LGALS1, CCND2, HEY1, RNF19A, GRAP2, FTH1, ME1, TNFRSF8, DACT1, RALGPS2, PRKCH, SYK, IL15, ACTN1, VASH2, FRMD4A, IRAK3, INPP4B, PTP4A3, PRKD3, FSCN1, CRY1, RIN3, ADAM8, TNIK, NAIP, KANK1, HSPA4L, STK39, ARNTL2, INAVA, DRAM1, CTNNA1, FLNA, APOL3, S100A10, APOBEC3C, GADD45B, TNFSF14, APOBEC3G, RDX, DNAJC12, PARVB, PTPN13, ASS1, SPART, THEMIS2, DLG5, GNAQ, BHLHE40, PIR, TRIP10, NMT2, VIM, FERMT2, GSDME, APOBEC3B

	focal adhesion	12	6.741573034	8.81E-04	TPM4, ACTN1, RDX, CTNNA1, SLA, FLNA, PARVB, VIM, WASF1, LPP, FERMT2, CORO1C
Molecular Function (MF)	cytokine receptor activity	4	2.247191011	0.015834092	EBI3, IL12B, IL6ST, IL27RA
	interleukin-27 receptor activity	2	1.123595506	0.018111084	IL6ST, IL27RA
	protein kinase A binding	3	1.685393258	0.021517762	RDX, WASF1, DACT1
	G-protein coupled receptor binding	4	2.247191011	0.02693575	APP, GNA15, GNAQ, FLNA
	ephrin receptor binding	3	1.685393258	0.032192345	APP, EFNA5, PIK3CG

Supplementary Table 11 Top 5 GO Enrichment Results for Upregulated DEGs Identified for the DCBCL vs CLL Comparison.

Category	Term	Count	%	P Value	Genes
Biological Process (BP)	inflammatory response	5	12.82051282	0.004091432	BCL6, CD180, STK39, PIK3CG, NAIP
	protein phosphorylation	5	12.82051282	0.008164566	SYK, PRKD3, STK39, PRKACB, PIK3CG
	innate immune response	5	12.82051282	0.015285099	SYK, CD180, SLA, PIK3CG, NAIP
	cell activation	2	5.128205128	0.020652161	SYK, CD24
	actin cytoskeleton organization	3	7.692307692	0.033935762	KANK1, BCL6, WASF1
Cellular Component (CC)	RNA polymerase II transcription factor complex	3	7.692307692	0.016324012	MXI1, BACH2, S100A10
Molecular Function (MF)	protein serine/threonine kinase activity	5	12.82051282	0.005733488	SYK, PRKD3, STK39, PRKACB, PIK3CG

Supplementary Table 12 Top 5 GO Enrichment Results for Downregulated DEGs Identified for the DCBCL vs CLL Comparison.

Category	Term	Count	%	P Value	Genes
Biological Process (BP)	immune response	17	12.23021583	3.46E-07	IFITM3, IFITM2, CCL22, TNFSF14, IL1R1, IL15, CCL20, GEM, IL27RA, RGS1, IL7, CCL5, FTH1, CCL4, TNFSF9, ACKR3, TNFRSF14
	cellular response to tumor necrosis factor	10	7.194244604	8.69E-07	VCAM1, CCL22, CCL20, CCL5, CCL4, CYP1B1, CD58, YBX3, ASS1, GSDME
	cellular response to interferon-gamma	8	5.755395683	6.04E-06	CCL22, CCL20, CCL5, CCL4, IL12B, CD58, VIM, ASS1
	positive regulation of ERK1 and ERK2 cascade	10	7.194244604	3.37E-05	APP, BMP2, CCL22, CCL20, CCL5, CCL4, SPRY2, ACKR3, PDE8A, FERMT2
	lymphocyte chemotaxis	5	3.597122302	8.96E-05	CCL22, CCL20, CCL5, CCL4, ADAM8
Cellular Component (CC)	plasma membrane	65	46.76258993	9.90E-08	IFITM3, KCNK5, APP, OXTR, SPARC, IFITM2, TUSC3, AQP9, TSPAN12, IL27RA, ADGRE1, RGS1, GRAP2, ME1, TNFRSF8, MUC13, PRKCH, CD300A, IL1R1, ACTN1, IRAK3, CKAP4, GEM, ANO3, FCER2, PTP4A3, MAP1B, ADAM8, IL6ST, STEAP1, COLEC12, PON2, EBI3, EFNA5, PLD1, GNA15, CTNNA1, FLNA, ANO10, TNFRSF14, CD58, BCHE, P2RY10, VCAM1, TNFSF14, RDX, CYBRD1, PARVB, PTPN13, SPART, BMP2, P2RX4, DLG5, GNAQ, TRIP10, SPRY2, RAB9A, TNFSF9, ACKR3, NMT2, VIM, ITM2A, FERMT2, GSDME, ITM2C
	cytoskeleton	17	12.23021583	6.91E-07	TMOD1, FHOD3, TPM4, RDX, VASH2, FRMD4A, PARVB, PTPN13, CKAP4, SEPTIN8, FSCN1,

					TRIP10, CTNNA1, SPRY2, TNIK, VIM, FERMT2
	cytoplasm	58	41.72661871	1.77E-04	FHOD3, APP, SPARC, IFI35, ARHGAP6, YBX3, MEOX1, HERC5, TUBB6, LGALS1, CCND2, HEY1, RNF19A, GRAP2, FTH1, ME1, TNFRSF8, DACT1, PRKCH, IL15, ACTN1, VASH2, FRMD4A, IRAK3, INPP4B, PTP4A3, FSCN1, CRY1, RIN3, ADAM8, TNIK, HSPA4L, ARNTL2, INAVA, DRAM1, CTNNA1, FLNA, APOL3, APOBEC3C, GADD45B, TNFSF14, APOBEC3G, RDX, PARVB, PTPN13, ASS1, SPART, THEMIS2, DLG5, GNAQ, BHLHE40, PIR, TRIP10, NMT2, VIM, FERMT2, GSDME, APOBEC3B
	endoplasmic reticulum lumen	10	7.194244604	2.15E-04	BCHE, APP, LGALS1, IGFBP4, EBI3, IL12B, TIMP1, APOL1, CKAP4, FBN1
	extracellular exosome	30	21.58273381	2.79E-04	APP, TUBB6, LGALS1, FTH1, FLNA, TNFRSF8, CD58, TIMP1, PDE8A, PRKCH, VCAM1, TPM4, CD300A, ACTN1, RDX, CYBRD1, PTPN13, CKAP4, ASS1, FCER2, P2RX4, GNAQ, FSCN1, TRIP10, RAB9A, TNIK, VIM, IL6ST, BLVRA, ITM2C
Molecular Function (MF)	cytokine activity	9	6.474820144	7.59E-05	BMP2, TNFSF14, IL15, IL7, EBI3, CCL4, TNFSF9, IL12B, TIMP1
	actin filament binding	9	6.474820144	2.33E-04	TMOD1, FHOD3, TPM4, ACTN1, FSCN1, CTNNA1, FLNA, FERMT2, CORO1C
	cytokine binding	5	3.597122302	3.04E-04	EBI3, IL12B, TNFRSF14, IL6ST, IL27RA
	protein binding	109	78.41726619	7.67E-04	FHOD3, IFITM3, APP, SPARC, AQP9, IFI35, IL27RA, HERC5, TUBB6, LGALS1, CCND2, RNF19A, GRAP2, FTH1, IL12B, CYP1B1, IER3, PRKCH, TPM4, CD300A, IGFBP4, IL1R1, ACTN1, BOLA1,

					ZBTB32, VASH2, GEM, INPP4B, MAP1B, FSCN1, RIN3, ADAM8, TNIK, IL6ST, EBI3, EFNA5, PLD1, INAVA, CORO1C, NACC2, TNFRSF14, MSC, PPP1R26, APOBEC3C, GADD45B, TNFSF14, APOBEC3G, RDX, CYBRD1, PARVB, SPART, BMP2, LHX2, IL7, DLG5, GNAQ, BHLHE40, PIR, UNC119, NMT2, ITM2A, FERMT2, APOBEC3B, GSDME, FBN1, ITM2C, PRR5, KCNK5, TSPAN12, YBX3, MEOX1, SEC61A1, HEY1, SIX3, ME1, TIMP1, DACT1, IL15, IRAK3, CKAP4, DDB2, FCER2, PTP4A3, CRY1, BLVRA, COLEC12, DRAM1, RXRA, CCL5, LMNA, CCL4, CTNNA1, FLNA, CD58, APOL1, BCHE, CCL22, CCL20, PTPN13, ASS1, SEPTIN8, P2RX4, THEMIS2, TRIP10, SPRY2, RAB9A, ACKR3, VIM, COPG1
	CCR chemokine receptor binding	4	2.877697842	0.001159785	CCL22, CCL20, CCL5, CCL4

Supplementary Table 13 The DEGs with the top 10 highest network degree values for PPI networks generated for the DLBCL vs CLL comparison.

Node	IL15	CCL4	FLNA	CCL5	IL7	VCAM1	SYK	ACTN1	CCL20	IL1R1
Description	down	down	down	down	down	down	up	down	down	down
Degree	30	26	24	24	24	24	20	18	18	18

Supplementary Table 14 The upregulated DEGs with the top 10 highest network degree values for PPI networks generated for the DLBCL vs CLL comparison**.**

Node	ANKMY2	PIK3CG	BZW2	BACH2	BCL6	ZHX2	SYK	CD72	CD180	SLA
Description	up	up	up	up	up	up	up	up	up	up
Degree	8	8	6	4	4	2	2	2	2	2

Supplementary Table 15 The downregulated DEGs with the top 10 highest network degree values for PPI networks generated for the DLBCL vs CLL comparison**.**

Node	IL15	VCAM1	FLNA	CCL5	CCL4	IL7	CCL20	IL1R1	ACTN1	APP
Description	down	down	down	down	down	down	down	down	down	down
Degree	26	24	22	22	22	20	18	18	16	16

Supplementary Table 16 Primer sequences for real - time quantitative PCR.

Primer		Sequence (5‘-3’)
GAPDH (human)	Forward	TGCACCACCAACTGCTTAG
	Reverse	AGAGGCAGGGATGATGTTC
IL15(human)	Forward	TGTTAGCAGATAGCCAGCCC
	Reverse	CTGCACTGAAACAGCCCAAA
PIK3CG (human)	Forward	TGGATATGAAGGGAGCCCCA
	Reverse	CATGCCCTATGCGACCTGAT

Supplementary Table 17 The relative expression level of IL15

	Normal	CLL	DLBCL
sample1	1.00	0.75	0.40
sample2	0.15	0.54	0.38
sample3	0.37	0.92	0.19
average	0.50	0.74	0.32
P value		CLL vs DLBCL=0.0176	

Supplementary Table 18 The relative expression level of PIK3CG

	Normal	CLL	DLBCL
sample1	1.00	1.38	2.23
sample2	0.90	0.46	4.02
sample3	1.76	0.36	3.09
average	1.22	0.73	3.11
P value		CLL vs DLBCL=0.0301	

Supplementary Table 19 Abbreviations.

Abbreviation	Full name
DLBCL	Diffuse large B-cell lymphoma
CLL	chronic lymphocytic leukemia
NHL	non-Hogkin lymphoma
DEG	differentially expressed genes
PPI	protein-protein interaction
GO	Gene Ontology
ALB	albumin
TT	thrombin time
WBC	White Blood Cell
TG	triglycerides
HDL	high/low-density lipoprotein
GGT	glutamyl transpeptidase
ALP	alkaline phosphatase
ALT	alanine transaminase
NEU%	neutrophil percentage
NLCs	nurse-like cells
IGHV	immunoglobulin heavy chain
TNF	tumor necrosis factor
GEO	Gene Expression Omnibus

BP	biological process
CC	cellular component
MF	molecular function
PLT	platelets
HGB	hemoglobin
NEU #	absolute neutrophil count
LYMPH%	lymphocyte percentage
LYMPH #	absolute lymphocyte count
TP	total protein
GLB	globulin
AST	aspartate transaminase
TBA	total bile acid
TBIL	total bilirubin
DBIL	direct bilirubin
CREA	creatinine
UA	uric acid
LDH	lactate dehydrogenase
CK	creatine kinase
CK-MB	CK isoenzyme
a-HBDH	5-hydroxybutyrate dehydrogenase
AMY	amylase

K	potassium
Na	sodium
Cl	chlorine
Ca	calcium
P	phosphorus
Mg	magnesium
CO2CP	carbon dioxide combining power
GLU	glucose
TC	total cholesterol
FA	folic acid
B12	vitamin B12
F	ferritin
TPO	erythropoietin
UIBC	unsaturated iron
TIBC	total iron binding force
ISAT	iron saturation
PT	prothrombin time
INR	international standardized ratio
APTT	partial thromboplastin time
FIB	fibrinogen
ATI I	antithrombin III activity

FDB	fibrinogen decomposition products
DD	D-dimer quantification
IgG	immunoglobulin G
IgA	immunoglobulin A
Ig	immunoglobulin M
C3	complement C3
C4	complement C4
CRP	C-reactive protein
ASO	rheumatoid factor Anti Streptolysin O
VIP	variable importance
SE	standard error
KEGG	Kyoto Encyclopedia of Genes and Genomes

References

1. Stone N, Kendall C, Smith J, Crow P, Barr H. Raman Spectroscopy for Identification of Epithelial Cancers. *Faraday Discuss* (2004) 126:141-57; discussion 69-83. doi: 10.1039/b304992b

2. González-Solís JL, Martínez-Espinosa JC, Salgado-Román JM, Palomares-Anda P. Monitoring of Chemotherapy Leukemia Treatment Using Raman Spectroscopy and Principal Component Analysis. *Lasers Med Sci.* (2014) 29(3):1241-9. doi: 10.1007/s10103-013-1515-y

3. Nargis HF, Nawaz H, Ditta A, Mahmood T, Majeed MI, Rashid N, et al. Raman Spectroscopy of Blood Plasma Samples from Breast Cancer Patients at Different Stages. *Spectrochim Acta A Mol Biomol Spectrosc.* (2019) 222:117210. doi: 10.1016/j.saa.2019.117210

4. Pully VV, Lenferink ATM, Otto C. Time Lapse Raman Imaging of Living Cells. *J Raman Spectrosc* (2015) 42:23.

5. Managò S, Valente C, Mirabelli P, Circolo D, Basile F, Corda D, et al. A Reliable Raman-Spectroscopy-Based Approach for Diagnosis, Classification and Follow-up of B-Cell Acute Lymphoblastic Leukemia. *Sci Rep.* (2016) 6:24821. doi: 10.1038/srep24821

6. da Silva AM, de Siqueira EOFSA, de Brito PL, Silveira L. Spectral Model for Diagnosis of Acute Leukemias in Whole Blood and Plasma through Raman Spectroscopy. *J Biomed Opt.* (2018) 23(10):1-11. doi: 10.1117/1.Jbo.23.10.107002
